# Design, Synthesis and Cancer Cell Growth Inhibition Evaluation of New Aminoquinone Hybrid Molecules

**DOI:** 10.3390/molecules24122224

**Published:** 2019-06-14

**Authors:** Andrea Defant, Ines Mancini

**Affiliations:** Laboratory of Bioorganic Chemistry, Department of Physics, University of Trento, Via Sommarive 14, 38123 Trento, Italy; andrea.defant@unitn.it

**Keywords:** hybrid molecules, quinolinequinones, naphtoquinones, 3,4,5-trimethoxyphenyl group, docking calculation, antitumor activity, cytotoxicity

## Abstract

Molecular hybridization has proven to be a successful multi-target strategy in the design and development of new antitumor agents. Based on this rational approach, we have planned hybrid molecules containing covalently linked pharmacophoric units, present individually in compounds acting as inhibitors of the cancer protein targets tubulin, human topoisomerase II and ROCK1. Seven new molecules, selected by docking calculation of the complexes with each of the proteins taken into consideration, have been efficiently synthesized starting from 2,3-dichloro-1,4-naphtoquinone or 6,7-dichloro-5,8-quinolinquinone. By screening the full National Cancer Institute (NCI) panel, including 60 human cancer cell lines, four molecules displayed good and sometimes better growth inhibition GI_50_ than the ROCK inhibitor Y-27632, the Topo II inhibitor podophyllotoxin and the tubulin inhibitor combretastatin A-4. The relative position of *N,N* heteroatoms in the structures of the tested compounds was crucial in affecting bioactivity and selectivity. Furthermore, compound **3** (2-(4-(2-hydroxyethyl)piperazin-1-yl)-3-(3,4,5-trimethoxyphenoxy)naphthalene-1,4-dione) emerged as the most active in the series, showing a potent and selective inhibition of breast cancer BT-549 cells (GI_50_ < 10 nM).

## 1. Introduction

Cancer is one of the prominent causes of death worldwide, and there is an urgent need to introduce new therapeutic agents due to resistance and severe side effects shown by the currently available drugs. Using molecules to act on different tumor targets simultaneously has shown a higher therapeutic potential than single-target chemotherapy. In the recent rational design of anticancer drugs, molecular hybridization is a promising approach. A hybrid molecule usually contains two or more pharmacophore scaffolds present in single therapeutically active agents, connected together in a new single structure by covalent bonds. The selection of these moieties is based on the strategy of combining structures and pharmacological activities of known drugs and bioactive natural or synthetic compounds [1]. The aim of this strategy is to obtain new therapeutic agents that are able not only to reduce undesirable side effects of the parent drugs, but also to display a modified selectivity profile, a higher affinity and a better therapeutic effect than the administration of a combination of the single-target drugs [2]. Hybrid molecules have demonstrated more favorable pharmacokinetic and pharmacodynamic parameters, in addition to dual or multiple modes of action, due to their ability to inhibit more than one biological target [1]. It is therefore understandable that the investigation of new hybrid anticancer drugs has recently become of great therapeutic interest.

Tubulin is a protein composed of microtubules which are the main components of the cellular cytoskeleton. They play a pivotal role in proliferation, migration and mitosis. Molecules able to bind this protein interfere with microtubule polymerization and depolymerization, inducing cell cycle arrest and leading to apoptosis in cancer cells. A variety of molecules have been proven to act efficiently as tubulin inhibitors, but their therapeutic use is limited by toxicity and development of resistance. Molecular hybridization has been used with good results to target this protein [3].

Topoisomerases (Topo I and Topo II) are enzymes that change the topological state of DNA through the breaking and rejoining of DNA strands. They have a relevant role in replication, recombination, transcription and preservation of genome stability. A series of currently used anticancer drugs and molecules in clinical trials act as inhibitors of these enzymes, stabilizing the DNA-Topo complex by intercalation between DNA base pairs. Therefore, inhibition of human topoisomerases is a promising target in the development of new antitumor agents [4].

Rho-associated kinases, known as two isoforms, ROCK1 and ROCK2, have been shown to induce stress fiber formation, cancer cell migration and metastasis. ROCK protein expression is elevated in several types of cancer. In a series of both in vitro and in vivo studies, advantages have been demonstrated by blocking these proteins, obtaining results especially in reducing tumor growth and in preventing metastases. The data support the potential of ROCKs as targets against tumors [5].

We report here on the efficient synthesis of seven new amino-quinone derivatives, designed as hybrid molecules that combine structural units present in inhibitors of tubulin, topoisomerase II and ROCK as tumor targets, and selected by docking calculations as ligands of these proteins. The evaluation on the growth inhibition of cancer cells by the in vitro NCI screening has been related to the structural features of these hybrid molecules.

## 2. Results and Discussion

### 2.1. In Silico Molecular Modeling

**Figure 1:**
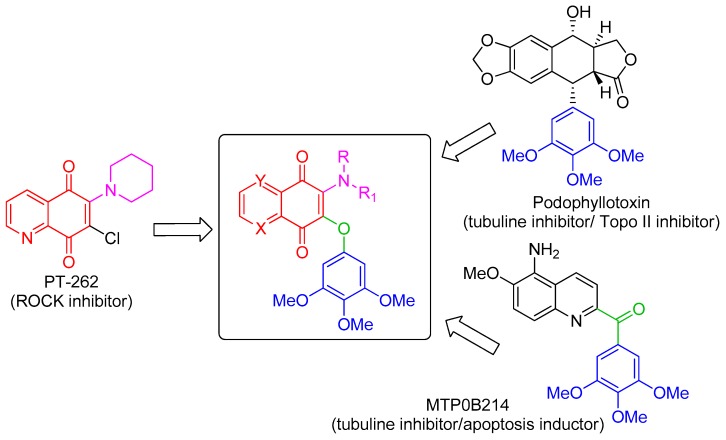
Design strategy of the target hybrid molecules.

The 3,4,5-trimethoxyphenyl (TMP) unit is a structural feature of anti-neoplastic molecules. It is the case of the natural phenols combretastatins, whose biological activities have been attributed to the presence of this moiety’s ability to target tubulin by the inhibition of microtubule formation. Other representative examples are given by colchicine and podophyllotoxin (Figure 1) [6], which make this pharmacophore a peculiar structural motif among the effective tubulin inhibitors studied in the last decades, with a special value in the design of new antitumor drugs. In addition, podophyllotoxin derivatives acting as topoisomerase II inhibitors are drugs commonly used in clinical oncology [7]. MPT0B214, also showing the TMP unit (Figure 1), inhibits tubulin polymerization and induces apoptosis through mitochondria-mediated pathways [8]. Naphthoquinone and quinolinequinones cores are other representative chemical scaffolds for the development of antitumor agents, which are present in structures of natural and synthetic bioactive products [9,10]. Their molecular mechanism includes reactive oxygen species (ROS) generation mediated by naphtoquinone oxidoreductase 1 (NQO1) bioreduction [11]. Quinolinequinone is a structural unit also present in 7-chloro-6-piperidinyl-quinoline-5,8-dione (PT-262, Figure 1), a synthetic molecule showing an effective inhibition of ROCK kinase activities [12].

**Scheme 1:**
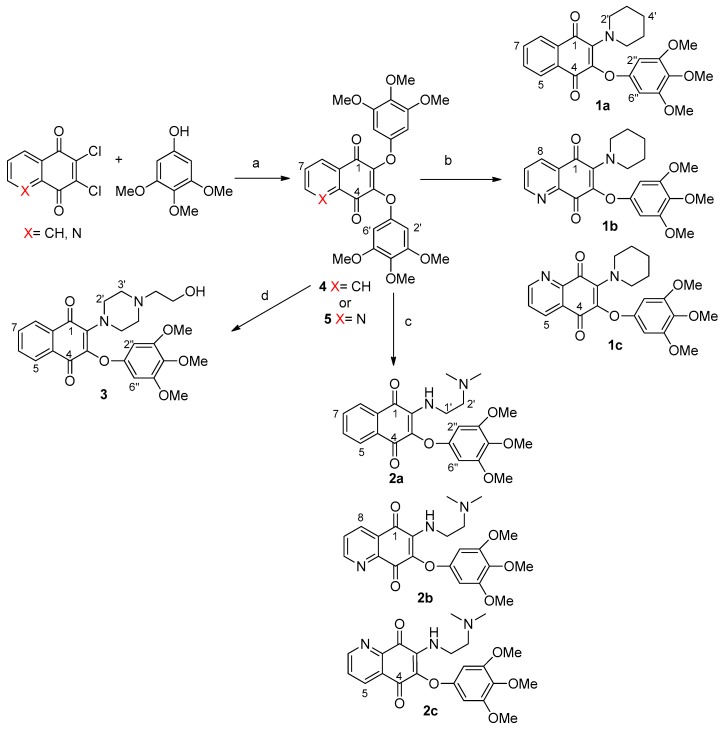
Synthesis of molecules **1a**–**c, 3a**–**c** and **3.** Reagents and conditions: (**a**) K_2_CO_3_, DMSO, r.t. 48 h; (**b**) piperidine, CH_2_Cl_2_, r.t. 24 h; (**c**) *N1*,*N1*-dimethylethane-1,2-diamine, CH_2_Cl_2_, r.t. 24 h; (**d**) 2-(piperazin-1-yl)ethan-1-ol, CH_2_Cl_2_, r.t. 24 h. Arbitrary numbering is for convenience.

Based on these evidences, the structures considered in this molecular hybridization design show a quinone unit with X, Y as CH or *N*, substituted by both a linear or cyclic amine and a 3,4,5 trimethoxyphenyl ether (Figure 1). A very good overlap has been observed for the energy-minimized structures of PT-262 and podophyllotoxin in cases where quinolinequinone is substituted by piperidine as a cyclic amine (corresponding to **1b** in Scheme 1) in the planned molecule, showing an *N,N*-anti configuration (as in PT-262 (Figure S1)).

In silico screening of the interactions with the target proteins tubulin, human topoisomerase II β and human ROCK1 (Table S1) via docking calculation permitted the selection of the new molecules **1a**–**c**, **2a**–**c** and **3** (Scheme 1). Their drug-likeness was computationally predicted regarding the physico-chemical properties relevant for the development of a drug. By using the free web tool SwissADME, a series of descriptors were calculated, taking into account lipophilicity, size, polarity, solubility, flexibility and unsaturation [13]. All the designed molecules showed a behavior that respected the parameters necessary for good bioavailability (Table S2).

### 2.2. Synthesis of Compounds 1a–c, 2a–c and 3

The desired products were easily accessible using the common precursors **4** and **5**, by the reaction carried out at room temperature in DMSO in the presence of potassium carbonate of 3,4,5-trimethoxyphenol with 2,3-dicholoro-1,4-naphtoquinone or 6,7-dichloro-5,8-quinolinequinone respectively (Scheme 1), via a proposed mechanism involving a Michael addition followed by chloride elimination [14]. The following treatment of compound **4** with piperidine, *N,N*dimethylethane-1,2 diamine or 2-(piperazin-1-yl)ethan-1-ol in dichloromethane at room temperature provided the products **1a**, **2a** and **3**, respectively, with global yields in the range 77% ÷ 79%, as evaluated after chromatographic purification of the products. It has been reported that similar quinones with one chlorine and one alkoxyl unit preferentially react by replacing the latter group [15,16], and therefore the use of the symmetric disubstituted 3,4,5-trimethoxyphenoxyl reagent was essential. Through a similar procedure, the products **1b**, **1c**, **2b** and **2c** were obtained starting from the quinolinequinone **5**. Pure regioisomers **1b** and **1c** with a 40:60 ratio resulted from chromatographic separation on silica gel by elution with dichloromethane/methanol/triethylamine 95:5:0.1. Similarly, **2b** and **2c** were isolated in a 42:58 ratio. Structural assignments of the regioisomer pairs **1b**/**2b** and **1c**/**2c** were based on long-range hetero-correlations observed by HMBC experiments. In detail, taking as references the data obtained for similar isomers [17]: (*i*) ^3^*J*(^1^H, ^13^C) couplings of the signal at 8.35 ppm for H-8 with 183.2 ppm for C(1)=O in **1b**, and 8.36 ppm with 182.0 ppm for **2b**, supported the *N,N*-anti configuration; (*ii*) ^3^*J*(^1^H, ^13^C) couplings of the signal at 8.34 ppm for H-5 with 177.1 ppm for C(1)=O in **1c**, and 8.38 ppm with 176.3 ppm for **2c**, supported the *N,N*-syn configuration (Scheme 1).

### 2.3. Biological Evaluation

Compounds **1a**–**c**, **2a**–**c** and **3** were subjected to in vitro growth percentage activity on the NCI full panel containing 60 human cancer cell lines at a single dose at 10 μM. From this evaluation compounds **1a**, **1c** and **2a** were considered inactive (Table 1), whereas **1b**, **2b**, **2c** and **3** were selected for further investigation at five concentration levels. Table 2 reports GI_50_ data (defined as the concentration values of the molecules inhibiting the growth of cancer cells by 50%) in comparison with the values reported in the NCI database for the ROCK inhibitor Y-27632, the Topo II inhibitor podophyllotoxin and the tubulin inhibitor combretastatin A-4 [18]. The GI_50_ values of the new tested compounds resulted in 10 nM ÷ 10 µM range.

**Figure 2:**
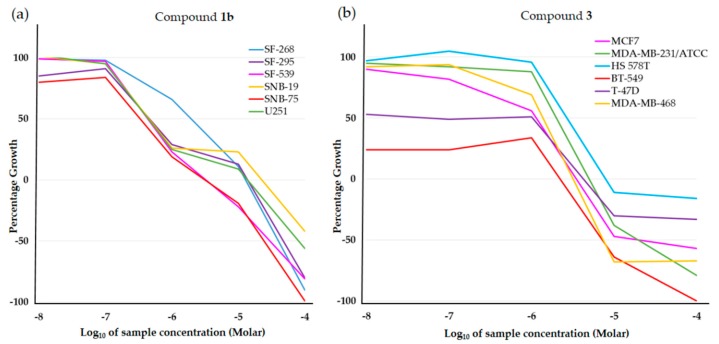
Dose response curves at NCI fixed protocol: (**a**) for compound **1b** on the indicated central nervous system (CNS) cancer cell lines, and (**b**) for compound **3** on the indicated breast cancer cell lines. The curves were obtained at five concentrations (log dilutions from 10^–4^ to 10^–8^ M). The concentrations of each compound, which inhibited 50% of cancer cell growth (GI_50_), were deduced and are the values reported in Table 1.

Submicromolar activities were observed for compound **1b** (Table 2), in particular against central nervous system (CNS) cancer for most of the tested cell lines (Figure 2a). Compound **2b** showed the highest inhibitions on MDA-MB-468 breast cancer cells (GI_50_ = 0.133 µM) and on both HCT-116 and SW-620 colon cell lines (GI_50_ 0.277 and 0.318 µM, respectively, Table 2). Notably, the relative structural position of *N*,*N* heteroatoms is crucial in affecting bioactivity and selectivity. In fact, compared with **2b**, its *N*,*N*-syn regioisomer **2c** displayed a lower activity corresponding to a growth inhibition in the micromolar range on the same cancer cell lines. A more pronounced disparity was observed for the pair **1b**/**1c**, where the *N*,*N*-syn isomer **1c** showed no activity. This is evident taking into account the values observed for all the synthesized compounds reported in Table 1 when expressed as the mean dose percentage of single high doses (10*^−^*^5^ M) from the full NCI 60 cell panel. Moreover, the structures lacking of the N atom on quinone unit as 1a and 2a provided inactive compounds (Table 1), whereas product 3 having additional heteroatoms on the C-2 substituent (specifically an NCH_2_CH_2_OH moiety) when compared with the inactive 1a, emerged as the most active molecule. Product **3** displayed submicromolar inhibition and was about 160 and 100 times more active than podophyllotoxin and combretastatin A-4, respectively, on T-47D breast cancer lines (Table 2). Furthermore, **3** emerged as the most promising in the series, with a selective GI_50_ value lower than 10 nM on BT-549 breast cancer cell line (Figure 2b).

## 3. Materials and Methods

### 3.1. Chemistry

#### 3.1.1. General

All reagents were purchased from Sigma Aldrich and used without further purification. Preparation of 6,7-Dichloroquinoline-5,8-dione followed a reported procedure [14,19]. The reaction yields were calculated for the products after chromatographic purification. Thin layer chromatography (TLC): Merck silica gel F_254_ or reversed phase Merck RP-18 F_254_, with visualization using UV light. Flash chromatography (FC): Merck Si 15–25 μm. Preparative thin layer chromatography (PLC): 20 × 20 cm Merck Kieselgel 60 F_254_ 0.5-mm plates. NMR spectra were recorded on a Bruker-Avance 400 spectrometer using a 5-mm BBI probe ^1^H at 400 MHz and ^13^C at 100 MHz in CDCl_3_ (relative to δ_H_ 7.25 and δ_C_ 77.00 ppm), with δ values in ppm and *J* values in Hz; assignments were supported by heteronuclear single quantum correlation (HSQC) and heteronuclear multiple bond correlation (HMBC) experiments. Electrospray ionization (ESI)-MS mass spectra were recorded using a Bruker Esquire-LC spectrometer by direct infusion of a methanol solution (source temperature 300 °C, drying gas N_2_, 4 L min^−1^, scan range *m/z* 100 ÷ 1000). Electron ionization (EI) mass spectra (*m/z*; rel%) and high resolution (HR)-EI data were recorded with a Kratos-MS80 mass spectrometer, heating at 213 °C for **2a**–**c**, at 276 °C for **1a**–**c** and at 417 °C for compound **3**, using a home-built computerized acquisition software.

#### 3.1.2. Typical Reaction Procedure for Precursors **4** and **5**

Anhydrous potassium carbonate (414 mg, 3.00 mmol, 1.5 equiv) was added to a solution of 2,3-dichloro-1,4-naphtoquinone (227 mg, 1.00 mmol, 1.0 equiv) or 6,7-dichloro-5,8-quinolinquinone (228 mg, 1.00 mmol, 1.0 equiv) and 3,4,5-trimethoxyphenol (384 mg, 2.00 mmol, 2.00 equiv) in 2.5 mL of dry DMSO, and the reaction mixture was stirred at room temperature for 48 h. The mixture was decanted to remove inorganic salt, and partitioned between dichloromethane/water (X3). The combined organic extracts were washed with water, dried over anhydrous Na_2_SO_4_ and concentrated in vacuo to give a solid that was purified by silica gel FC eluting with hexane/EtOAc (from 9:1 to 6:4 v/v) for **4,** and with dichloromethane/methanol/triethylamine (96:4:0.1 v/v) for **5.**

*2,3-Bis(3,4,5-trimethoxyphenoxy)naphthalene-1,4-dione* (**4**). TLC (hexane: EtOAc = 1:1 v/v): R_f_ = 0.50. Light orange solid. Yield: 87%. ^1^H-NMR (400 MHz, CDCl_3_) δ 8.15 and 7.79 (two m, 2H each, H-5/H-8 and H-6/H-7), 6.11 (s, 4H, H-2′, H-6′, H-2′and H-6′), 3.75 (s, 6H, -OCH_3_), 3.72 (s, 12H, -OCH_3_). ^13^C-NMR (100 MHz, CDCl_3_) δ 180.4 (C=O), 153.7, 134.7, 134.5, 130.7, 126.5, 94.4, 60.7 (-OCH_3_), 56.0(-OCH_3_). Significant HMBC correlations: 8.15 ppm with 180.4 ppm; 7.79 ppm with 130.7 ppm. ESI(+)-MS: *m/z* 545 [M + Na]^+^. HRMS(EI) calcd. for C_28_H_26_O_10_, 522.15260, found 522.15248.

*6,7-Bis(3,4,5-trimethoxyphenoxy)quinoline-5,8-dione* (**5**). TLC (CH_2_Cl_2_/MeOH = 95:5 v/v, with two drops of Et_3_N): R_f_ = 0.90. Brown solid. Yield: 93%. ^1^H-NMR (400 MHz, CDCl_3_) δ 9.11 (br d, *J* = 4.9 Hz, 1H, H-6), 8.49 (d, *J* = 7.5 Hz, 1H, H-8), 7.75 (dd, *J* = 7.5, 4.9 Hz 1H, H-7), 6.12 and 6.08 (two s, 2H each, H-2′, H-6′, H-2′and H-6′), 3.76 (s, 6H, -OCH_3_), 3.73 (s, 12H, -OCH_3_). ^13^C-NMR (100 MHz, CDCl_3_) δ 179.7 (C=O), 178.8 (C=O), 153.6, 153.5, 152.4, 146.7, 144.7, 135.5, 134.6, 127.8, 127.7, 94.0, 60.2 (-OCH_3_), 55.5 (-OCH_3_). Significant HMBC correlations: 9.11 ppm with 146.7 ppm; 8.49 ppm with 179.7 ppm; 7.75 ppm with 127.7 ppm; 6.12 and 6.10 ppm with 152.4, 146.7 and 134.6 ppm; 3.76 ppm with 134.6 ppm; 3.73 ppm with 152.4 ppm ESI(+)-MS: *m/z* 524 [M + H]^+^, 545 [M + Na]^+^, 562 [M + K]^+^. HRMS(EI) calcd. for C_27_H_25_NO_10_, 523.14785, found 523.14778.

#### 3.1.3. Typical Reaction Procedure for the Synthesis of Compounds **1a**–**c**, **2a**–**c** and **3**

A mixture of compound **4** or **5** (52.2 mg, 0.10 mmol, 1.0 equiv) and the suitable amine (0.20 mmol, 2.0 equiv) in 2 mL of anhydrous dichloromethane was stirred at room temperature for 24 h. The solvent was removed in vacuo and the residue was purified by PLC eluting with hexane/EtOAc 1:1 (v/v) for **1a** and CH_2_Cl_2_/MeOH/Et_3_N 95:5:0.1 (v/v) for the other compounds.

*2-(Piperidin-1-yl)-3-(3,4,5-trimethoxyphenoxy)naphthalene-1,4-dione* (**1a**). TLC (hexane: EtOAc = 1:1 v/v): Rf = 0.74. Red solid. Yield: 91%. ^1^H-NMR (400 MHz, CDCl_3_) δ 8.02 and 7.65 (two m, 2H each, H-5/H-8 and H-6/H-7), 6.18 (s, 2H, H-2′ and H-6′), 3.77 (s, 9H, three –OCH_3_), 3.43 (br s, 4H, H-2′and H-6′), 1.65 (br s, 6H, H-3′, H-4′and H-5′). ^13^C-NMR (100 MHz, CDCl_3_) δ 184.1 and 178.4 (two C=O), 154.3, 142.2, 134.2, 133.3, 132.3, 126.2, 96.2, 56.2 (-OCH_3_), 51.4, 28.2. Significant HMBC correlations: 8.02 ppm with 184.1 and 178.4 ppm; 7.65 ppm with 132.3 ppm. ESI(+)-MS: *m/z* 462 [M + K]^+^, 446[M + Na]^+^, 424 [M + H]^+^. HRMS(EI) calcd. for C_24_H_25_NO_6_, 423.16819, found 423.16810.

*6-(Piperidin-1-yl)-7-(3,4,5-trimethoxyphenoxy)quinoline-5,8-dione* (**1b**). TLC (CH_2_Cl_2_/MeOH = 95:5 v/v, with two drops of Et_3_N): R_f_ = 0.93. Violet solid. Yield: 40% (as single regioisomer). ^1^H-NMR (400 MHz, CDCl_3_) δ 8.97 (br d, *J* = 4.9 Hz, 1H, H-6), 8.35 (br d, *J* = 7.9 Hz, 1H, H-8), 7.59 (m, 1H, H-7), 6.20 (s, 2H, H-2′ and H-6′), 3.79 and 3.77 (two s, 9H, OCH_3_), 3.45 (m, 4H, H-2′ and H-6′), 1.65 (m, 6H, H-3′, H-4′and H-5′). ^13^C-NMR (100 MHz, CDCl_3_) δ 183.2 (C=O), 154.9, 154.0, 147.3, 145.4, 139.7, 135.7, 133.7, 128.5, 126.1, 91.8, 56.7, 51.4, 29.2, 24.0. Significant HMBC correlations: 8.35 ppm with 183.2 and 128.5 ppm; 7.59 ppm with 128.5 ppm; 6.20 ppm with 139.8 ppm; 3.45 ppm with 145.4 and 29.2 ppm. ESI(+)-MS: *m/z* 447 [M + Na]^+^. HRMS(EI) calcd. for C_23_H_24_N_2_O_6_, 424.16344, found 424.16329.

*7-(Piperidin-1-yl)-6-(3,4,5-trimethoxyphenoxy)quinoline-5,8-dione* (**1c**). TLC (CH_2_Cl_2_/MeOH = 95:5 v/v, with two drops of Et_3_N): R_f_ = 0.90. Violet solid. Yield: 60% (as single regioisomer). ^1^H-NMR (400 MHz, CDCl_3_) δ 8.93 (br d, *J* = 4.8 Hz, 1H, H-7), 8.34 (br d, *J* = 7.9 Hz, 1H, H-5), 7.61 (m, 1H, H-6), 6.19 (s, 2H, H-2′ and H-6′), 3.78 (s, 9H, OCH_3_), 3.50 (br s, 4H, H-2′ and H-6′), 1.69 (m, 6H, H-3′, H-4′ and H-5′). ^13^C-NMR (100 MHz, CDCl_3_) δ 182.4 and 177.1 (two C=O), 154.4 154.1, 151.6, 151.1, 147.7, 134.2, 133.5, 127.9, 92.9, 56.2, 52.6, 26.9, 25.0. Significant HMBC correlations: 8.93 ppm with 151.1 and 127.9 ppm; 8.34 ppm with 177.1 and 151.1 ppm; 7.61 ppm with 154.4 ppm; 6.19 ppm with 154.1 and 133.5 ppm. ESI(+)-MS: *m/z* 463 [M + K]^+^, 447 [M + Na]^+^, 425 [M + H]^+^. HRMS(EI) calcd. for C_23_H_24_N_2_O_6_, 424.16344, found 424.16328.

*2-((2-(Dimethylamino)ethyl)amino)-3-(3,4,5-trimethoxyphenoxy)naphthalene-1,4-dione* (**2a**). TLC (CH_2_Cl_2_/MeOH = 95:5 v/v, with two drops of Et_3_N): R_f_ = 0.30. Orange solid. Yield: 90%. ^1^H-NMR (400 MHz, CDCl_3_) δ 8.05 (d, *J* = 7.5 Hz, 2H, H-5 and H-8), 7.70 and 7.61 (two t, *J* = 7.7 Hz, 2H, H-6 and H-7), 6.40 (br s, 1H, *N*H), 6.22 (s, 2H, H-2′ and H-6′), 3.77 (s, 6H, two –OCH_3_), 3.75 (s, 3H, –OCH_3_), 3.56 (q, *J* = 5.5 Hz, 2H, H-1′), 2.49 (t, *J* = 5.5 Hz, 2H, H-2′), 2.22 (s, 6H, N(CH_3_)_2_). ^13^C-NMR (100 MHz, CDCl_3_) δ 182.4 and 177.8 (C=O), 155.1, 134.2, 133.3, 132.1, 129.8, 126.4, 92.6, 58.9 (C-2′), 55.9 (-OCH_3_), 44.6 (*N*(CH_3_)_2_), 41.7 (C-1′). Significant HMBC correlations: 8.05 ppm with 182.4 and 177.8 ppm; 7.65 ppm with 132.3 ppm; 7.70 ppm with 134.2 ppm; 7.61 ppm with 132.1 ppm; 6.22 ppm with 155.1 and 133.3 ppm. ESI(+)-MS: *m/z* 449 [M + Na]^+^, 427 [M + H]^+^. HRMS(EI) calcd. for C_23_H_26_N_2_O_6_, 426.17909, found 426.17896.

*6-((2-(Dimethylamino)ethyl)amino)-7-(3,4,5-trimethoxyphenoxy)quinoline-5,8-dione* (**2b**). TLC (CH_2_Cl_2_/MeOH = 95:5 v/v, with two drops of Et_3_N): R_f_ = 0.28. Orange solid. Yield: 42% (as single regioisomer). ^1^H-NMR (400 MHz, CDCl_3_) δ 8.99 (br d, *J* = 4.8 Hz, 1H, H-6), 8.36 (br d, *J* = 7.9 Hz, 1H, H-8), 7.55 (dd, *J* = 7.9, 4.8 Hz, 1H, H-7), 6.46 (br s, 1H, NH), 6.23 (s, 2H, H-2′and H-6′), 3.78 and 3.77 (two s, 9H, three –OCH_3_), 3.57 (m, 2H, H-1′), 2.49 (br t, *J* = 5.3 Hz, 2H, H-2′,), 2.22 (s, 6H, *N*(CH_3_)_2_). ^13^C-NMR (100 MHz,CDCl_3_) δ 182.0 (C=O), 176.1 (C=O), 154.6, 154.4, 148.6, 136.9, 133.7, 130.1, 123.8, 93.2, 57.8 (-OCH_3_), 57.7 (C-2′), 44.5 (-*N*(CH_3_)_2_), 41.1(C-1′). Significant HMBC correlations: 8.99 ppm with 123.8 ppm; 8.36 ppm with 182.0, 154.4 and 148.6 ppm; 7.55 ppm with 154.4 and 130.1 ppm; 6.24 ppm with 154.6 and 133.7 ppm; 3.78 and 3.77 ppm with 154.6 and 133.7 ppm; 2.49 ppm with 41.1 ppm; 2.23 ppm with 57.7 ppm. ESI(+)-MS: *m/z* 450 [M + Na]^+^, 428 [M + H]^+^. HRMS(EI) calcd. for C_22_H_25_N_3_O_6_, 427.17434, found 427.17446.

*7-((2-(Dimethylamino)ethyl)amino)-6-(3,4,5-trimethoxyphenoxy)quinoline-5,8-dione* (**2c**). TLC (CH_2_Cl_2_/MeOH = 95:5 v*/*v, with two drops of Et_3_N): R_f_ = 0.31. Orange solid. Yield: 58% (as single regioisomer). ^1^H-NMR (400 MHz, CDCl_3_) δ 8.92 (br d, *J* = 4.6 Hz, 1H, H-7), 8.38 (br d, *J* = 7.9 Hz, 1H, H-5), 7.62 (dd, *J* = 7.8, 4.5 Hz, 1H, H-6), 6.53 (br s, 1H, NH), 6.22 (s, 2H, H-2′ and H-6′), 3.78 (s, 9H, three –OCH_3_), 3.61 (m, 2H, H-1′), 2.50 (br t, *J* = 5.7 Hz, 2H, H-2′), 2.22 (s, 6H, *N*(CH_3_)_2_). ^13^C-NMR (100 MHz, CDCl_3_) δ 180.9 (C=O), 176.3 (C=O), 155.0, 153.2, 151.4, 147.2, 135.1, 133.6, 132.2, 127.0, 93.2, 58.5 (C-2′), 56.5 (-OCH_3_), 44.7 (-*N*(CH_3_)_2_), 41.2 (C-1′). Significant HMBC correlations: 8.92 ppm with 147.2, 135.1 and 127.0 ppm; 8.38 ppm with 180.9 (small), 176.3, 153.2 and 147.2 ppm; 7.62 ppm with 153.2, 135.1 and 132.2 ppm; 6.22 ppm with 155.0 and 133.6 ppm; 3.78 ppm with 155.0 and 133.6 ppm. ESI(+)-MS: *m/z* 450 [M + Na]^+^. HRMS(EI) calcd. for C_22_H_25_N_3_O_6_, 427.17434, found 427.17427.

*2-(4-(2-Hydroxyethyl)piperazin-1-yl)-3-(3,4,5-trimethoxyphenoxy)naphthalene-1,4-dione* (**3**). TLC (CH_2_Cl_2_/MeOH = 95:5 v/v, with two drops of Et_3_N): R_f_ = 0.30. Red solid. Yield: 88%. ^1^H-NMR (400 MHz, CDCl_3_) δ 8.01 and 7.66 (two m, 2H each, H-5/H-8 and H-6/H-7), 6.17 (s, 2H, H-2′ and H-6′), 3.77 (s, 9H, OCH_3_), 3.60 (m, 2H, CH_2_OH), 3.52 (m, 4H, H-2′), 2.59 (m, 4H, H-2′), 2.54 (m, 2H, *N*CH_2_). ^13^C-NMR (100 MHz, CDCl_3_) δ 183.7 and 178.6 (C=O), 154.0, 133.6, 133.5, 132.3, 131.7, 126.2, 92.6, 56.6, 55.9 (OCH_3_), 54.2, 49.8. Significant HMBC correlations: 8.01 ppm with 183.7 and 178.6 ppm; 7.66 ppm with 132.3 and 131.7 ppm; 6.17 ppm with 154.0 and 133.5 ppm. ESI(+)-MS: *m/z* 491 [M + Na]^+^, 469 [M + H]^+^. HRMS(EI) calcd. for C_25_H_28_N_2_O_7_, 468.18965, found 468.18951.

### 3.2. Computational Analysis

Calculations were carried out using Autodock Vina 1.1.2 [20], adopting a reported procedure [21]. The structures of human ROCK 1 (PDB ID: 2ETK), human topoisomerase II β (PDB ID: 3QX3) and tubulin (PDB ID: 5JCB) were determined by X-ray crystallography with a resolution of 2.9, 2.2 and 2.3 Å respectively. For the docking calculation, a grid box of 16 × 16 × 24 Å in x, y, z directions was created with a spacing of 1.00 Å, and centered at x = 51.964, y = 101.296, z = 29.213 for 2ETK; a grid box of 22 × 18 × 18 Å in x, y, z directions was created with a spacing of 1.00 Å, and centered at x = 32.884, y = 95.413, z = 50.785 for 3QX3; and a grid box of 18 × 28 × 26 Å in x, y, z directions was created with a spacing of 1.00 Å, centered at x = −13.614, y = 9.720, z = 20.911 for 5JCB. Results were expressed as energy associated to each ligand–enzyme complex in terms of Gibbs free energy values (Table S1). The visual ligand–enzyme interactions were displayed using Discovery Studio Visualizer v.19.1.0.18287 [22]. ADME predictions were performed using the online server Swiss-ADME [23].

### 3.3. Biological Evaluation

The synthesized compounds were evaluated for their in vitro activity against cancer cell lines by the National Cancer Institute (NCI-USA) following its anticancer drug development program based on automated sulforhodamine blue (SRB) cytotoxicity assay. The screening was a two-stage process, where after a first evaluation was carried out against the full panel of cell lines at a single dose of 10 μM, with the compounds exhibiting significant growth inhibition being tested at five concentration levels [24].

## 4. Conclusions

Based on the known benefits of considering a molecular hybridization approach in the design and development of new antitumor agents, we have planned new hybrid molecules containing pharmacophoric units, which present individually in compounds acting as inhibitors of the cancer protein targets tubulin, human topoisomerase II and ROCK1. Docking calculation of the complexes with each protein allowed us to select seven molecules, structurally characterized by a naphtoquinone or quinolinequinone moiety, and substituted by both a cyclic or functionalized amine and a 3,4,5-trimethoxyphenyl group.

The evaluation of human cancer cell inhibition by the seven synthetic compounds provided a qualitative structure–activity relationship study. What is more, compound **3** emerged as the most active in the series, displaying a selective nanomolar inhibition of breast cancer BT-549 cells. According to these promising findings and their easily accessible synthesis, the molecules herein reported are worthy of further biological investigation.

## Figures and Tables

**Table 1:** Mean dose percent values by one-dose assay (10^−5^ M) of all the tested compounds in the full NCI 60 cell panel.

Compound	Mean Growth Percent	Activity
**1a**	93.74	Inactive
**1b**	20.92	Active
**1c**	67.49	Inactive
**2a**	78.44	Inactive
**2b**	−4.33	Active
**2c**	12.79	Active
**3**	17.88	Active

**Table 2:** Inhibition of in vitro human cancer cell lines by compounds **1a**, **2b**, **2c** and **3**, in comparison with combretastatin A-4, podophyllotoxin and Y-27632 taken as reference compounds.

	Cytotoxicity GI_50_ (μM)						
	1b	2b	2c	3	Y-27632	Podophyllotoxin	Combretastatin A-4
**Cell lines**							
**Leukemia**							
CCRF-CEM	2.98	2.21	2.24	2.48	31.6	0.01	0.251
HL-60(TB)	1.76	0.811	1.43	1.66	100	0.01	0.01
K-562	0.354	0.659	2.90	2.19	100	-	0.316
MOLT-4	3.58	3.07	2.48	2.35	100	0.01	0.501
RPMI-8226	0.486	1.92	2.23	2.03	100	0.01	0.063
SR	1.89	3.17	2.13	2.87	25.1	0.01	1.99
**Non-Small Cell Lung Cancer**							
A549/ATCC	0.482	3.43	1.87	13.8	100	0.0126	0.020
HOP-92	10.4	1.88	2.01	1.46	1.26	0.0316	0.100
NCI-H226	0.506	16.67	2.08	2.92	100	0.01	0.251
NCI-H23	2.11	2.25	1.76	2.22	100	0.01	0.040
NCI-H322M	6.27	2.65	7.04	1.23	100	0.01	0.063
NCI-H460	2.77	3.11	1.65	3.13	100	0.01	0.050
NCI-H522	1.47	1.46	1.39	0.571	100	0.01	0.032
**Colon Cancer**							
COLO 205	2.79	1.10	2.44	1.80	100	0.01	6.31
HCC-2998	5.57	1.62	4.71	12.4	100	0.0126	0.158
HCT-116	0.513	0.277	1.85	1.44	100	0.01	0.079
HCT-15	0.442	2.07	2.78	1.56	100	0.0126	0.040
HT29	3.74	2.36	3.77	2.27	100	0.01	6.31
KM12	4.59	2.91	3.34	4.39	100	0.01	0.063
SW-620	1.37	0.318	1.55	1.79	100	0.01	0.063
**CNS Cancer**							
SF-268	1.94	2.39	2.87	1.73	63.1	0.01	0.063
SF-295	0.465	9.88	3.76	5.36	100	0.01	0.032
SF-539	0.434	1.78	1.85	2.48	100	0.01	0.025
SNB-19	0.450	2.00	2.86	3.41	100	0.01	0.025
SNB-75	0.334	1.89	1.39	1.68	10	0.01	1.259
U251	0.440	2.00	2.12	3.10	100	0.01	0.079
**Melanoma**							
LOX IMVI	1.01	0.508	1.76	1.81	100	0.01	0.050
MALME-3M	2.73	0.967	2.23	3.99	100	50.1	0.631
M14	0.552	1.84	3.07	1.96	100	0.0126	0.100
MDA-MB-435	1.11	1.72	1.53	1.76	100	0.01	0.010
SK-MEL-2	4.16	2.03	2.01	2.01	100	0.016	0.050
SK-MEL-28	1.35	-	2.00	2.34	100	0.01	5.012
SK-MEL-5	1.95	1.70	1.55	1.81	100	0.01	0.013
UACC-257	0.846	1.78	1.56	3.56	100	0.01	0.063
UACC-62	2.53	3.17	1.60	1.79	100	0.01	0.040
**Ovarian Cancer**							
OVCAR-3	1.77	0.364	2.02	1.02	79.4	0.0126	0.051
OVCAR-4	1.31	0.937	1.53	1.63	100	0.016	1.995
OVCAR-5	0.439	2.35	2.19	2.60	100	0.251	3.981
OVCAR-8	0.463	0.386	2.13	2.91	100	0.01	0.079
NCI/ADR-RES	1.06	7.40	5.95	3.19	100	0.01	0.063
SK-OV-3	3.28	11.6	7.49	5.31	100	0.01	0.251
**Renal Cancer**							
786-0	2.03	2.46	2.04	1.93	100	0.016	0.631
A498	0.640	2.17	2.09	2.04	100	0.01	0.100
ACHN	1.25	1.81	2.36	1.87	100	0.01	0.199
CAKI-1	0.595	2.10	2.51	3.59	100	0.1	0.251
RXF 393	0.694	2.00	2.83	1.55	100	0.01	0.398
SN12C	0.829	2.81	2.02	3.11	100	0.016	0.251
TK-10	3.27	3.63	3.91	4.97	10	0.0316	3.162
UO-31	1.18	1.80	1.29	1.61	100	0.016	1.000
**Prostate Cancer**							
PC-3	0.914	2.43	2.72	3.19	100	0.01	0.010
DU-145	2.60	3.43	4.19	-	100	0.01	0.013
**Breast Cancer**							
MCF7	0.509	0.304	1.20	1.15	100	0.01	0.010
MDA-MB-231/ATCC	4.14	2.55	1.81	2.00	100	0.01	0.016
HS 578T	0.415	3.06	2.84	2.69	100	0.01	0.010
BT-549	0.819	2.68	4.96	<0.01	100	0.01	0.020
T-47D	2.53	1.67	1.98	0.510	100	79.4	50.12
MDA-MB-468	1.77	0.133	0.285	1.37	100	0.01	0.079
**MGM** ^a)^	1.29	1.82	2.24	2.24	91.6	1.52	1.52

^a)^ MGM (mean graph medium) value as average GI50 (µM) over all cell lines investigated.

## Supplementary Materials

The following are available online. Table S1: Energy data from docking calculation by Autodock Vina for **1a**–**c**, **2a**–**c** and **3**, in comparison with original and reference ligands; Table S2: ADME prediction of **1a**–**c**, **2a**–**c**, **3** and reference compounds evaluated by on-line Server Swiss-ADME; Figure S1: Overlapping of the energy minimized structures **1b**, PT-262 and podophyllotoxin; Figures S2–S23: NMR spectra of compounds **1a**–**c**, **2a**–**c**, **3**, **4** and **5**.
