# Supplementary material for: Design, Synthesis and Cancer Cell Growth Inhibition Evaluation of New Aminoquinone Hybrid Molecules
Source: Molecules. 2019 Jun 14;24(12):2224. doi: 10.3390/molecules24122224 (PMC6630839; doi:10.3390/molecules24122224)
Supplement: Supplementary file 1 [file molecules-24-02224-s001.pdf]

Supplementary Material

Design, synthesis and cancer cell growth inhibition evaluation of new aminoquinone hybrid molecules

Andrea Defant [1] **and Ines Mancini** [1,*]

[1] Laboratory of Bioorganic Chemistry, Department of Physics, University of Trento, Via Sommarive 14, 38123 Trento, Italy; andrea.defant@unitn.it (A.D.); ines.mancini@unitn.it (I.M.)

*Correspondence: ines.mancini@unitn.it ; Tel.: +39-461-281-548

Table of Contents

Table S1. Energy data (in Kcal/mol) from docking calculation by Autodock Vina for the new ligand molecules **1a-1c**, **2a-2c** and **3**, in comparison with original and reference ligands.

Table S2. ADME Prediction of compounds **1a-c**, **2a-c**, **3** (molecules 1-7, respectively) and reference compounds evaluated by on-line Server Swiss-ADME.

Figure S1. Overlapping of the energy minimized structures **1b** (in red), PT-262 (in green) and podophyllotoxin (in blue). Hydrogen atoms are omitted for clarity.

Figure S2. ^{1}HNMR spectrum (400MHz, $CDCl_3$) of compound **1a.**

Figure S3. ^{1}H,^{13}C correlations by HSQC experiment (400MHz, $CDCl_3$) of **1a.**

Figure S4. ^{1}H,^{13}C long range correlations by HMBC experiment (400MHz, $CDCl_3$) of **1a.**

Figure S5. ^{1}HNMR spectrum (400MHz, $CDCl_3$) of compound **1b.**

Figure S6. ^{1}H,^{13}C correlations by HSQC experiment (400MHz, $CDCl_3$) of **1b.**

Figure S7. ^{1}H,^{13}C long range correlations by HMBC experiment (400MHz, $CDCl_3$) of **1b.**

Figure S8. ^{1}HNMR spectrum (400MHz, $CDCl_3$) of compound **1c.**

Figure S9. ^{1}H,^{13}C correlations by HSQC experiment (400MHz, $CDCl_3$) of **1c.**

Figure S10. ^{1}H,^{13}C long range correlations by HMBC experiment (400MHz, $CDCl_3$) of **1c.**

Figure S11. ^{1}HNMR spectrum (400MHz, $CDCl_3$) of compound **2a.**

Figure S12. ^{1}H,^{13}C correlations by HSQC experiment (400MHz, $CDCl_3$) of **2a.**

Figure S13. ^{1}H,^{13}C long range correlations by HMBC experiment (400MHz, $CDCl_3$) of **2a.**

Figure S14. ^{1}HNMR spectrum (400MHz, $CDCl_3$) of compound **2b.**

Figure S15. ^{1}H,^{13}C long range correlations by HMBC experiment (400MHz, $CDCl_3$) of **2b.**

Figure S16. ^{1}HNMR spectrum (400MHz, $CDCl_3$) of compound **2c.**

Figure S17. ^{1}H,^{13}C correlations by HSQC experiment (400MHz, $CDCl_3$) of **2c.**

Figure S18. ^{1}H,^{13}C long range correlations by HMBC experiment (400MHz, $CDCl_3$) of **2c.**

Figure S19. ^{1}HNMR spectrum (400MHz, $CDCl_3$) of compound **3.**

Figure S20. ^{1}H,^{13}C correlations by HSQC experiment (400MHz, $CDCl_3$) of **3.**

Figure S21. ^{1}H,^{13}C long range correlations by HMBC experiment (400MHz, $CDCl_3$) of **3.**

Figure S22. ^{1}HNMR spectrum (400MHz, $CDCl_3$) of precursor **4.**

Figure S23. ^{1}HNMR spectrum (400MHz, $CDCl_3$) of precursor **5.**

Table S1. Energy data (in Kcal/mol) from docking calculation by Autodock Vina for the new ligand molecules **1a**-**1c**, **2a**-**2c** and **3**, in comparison with original and reference ligands.

**Molecule**	**Structure**	**ΔE**<br>*5JCB*[a)]	**ΔE**<br>3QX3[b)]	**ΔE**<br>*2ETK*[c)]
**1a**	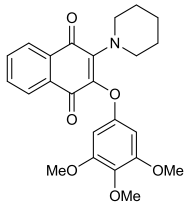	-8.3	-8.7	-8.2
**1b**	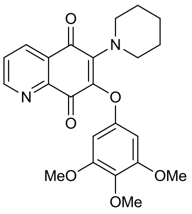	-8.3	-10.4	-7.8
**1c**	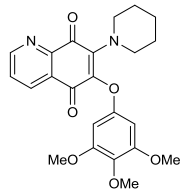	-8.3	-10.2	-8.0
**2a**	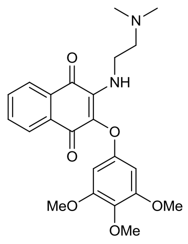	-.8.2	-9.0	-7.6
**2b**	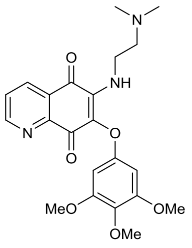	-7.9	-9.4	-6.9
**2c**	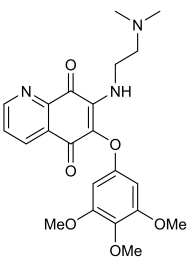	-8.1	-9.6	-6.7
**3**	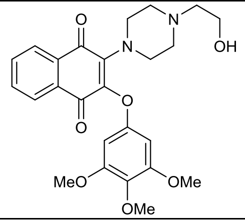	-8.4	-10.6	-7.8

PDB original ligands and reference molecules				
**Molecule**	**Structure**	**ΔE** *5JCB*[a)]	**ΔE** 3QX3[b)]	**ΔE** *2ETK*[a)]
**HFS** (original ligand)	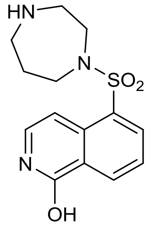	n.d.	n.d.	-8.1
**PT-262**	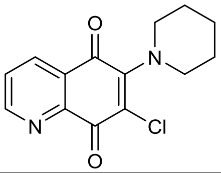	n.d.	n.d.	-7.3
**Y-27632**	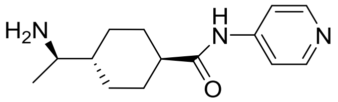	n.d.	n.d.	-6.7
**VP-16** (original ligand)	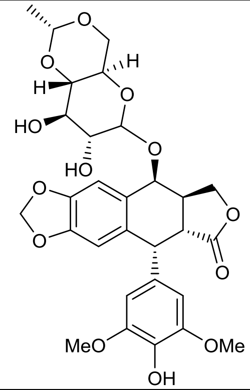	n.d.	-13.3	n.d.
**NV4** (original ligand)	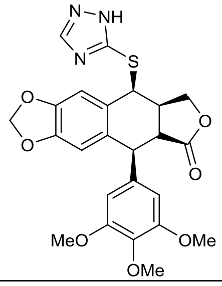	-10.3	n.d.	n.d.
**MTP**	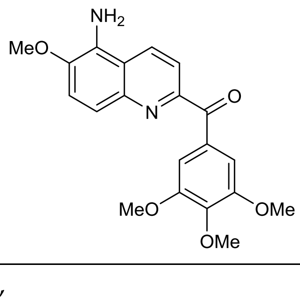	-8.1	n.d.	n.d.

[a)] tubulin (PDB ID: 5JCB),

[b)]human topoisomerase II β (PDB ID: 3QX3)

[c)] human ROCK 1 (PDB ID: 2ETK)

Table S2. ADME Prediction of compounds **1a-c**, **2a-c**, **3** (molecules 1-7, respectively) and reference compounds evaluated by online Server Swiss-ADME.

Molecule 1

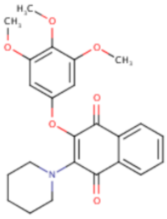

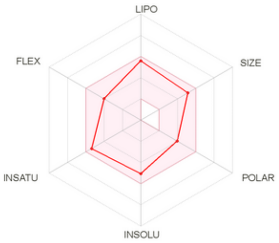

SMILES COc1cc(OC2=C(N3CCCCC3)C(=O)c3c(C2=O)cccc3)cc(c1OC)OC

Physicochemical Properties	
Formula	C24H25NO6
Molecular weight	423.46 g/mol
Num. heavy atoms	31
Num. arom. heavy atoms	12
Fraction Csp3	0.33
Num. rotatable bonds	6
Num. H-bond acceptors	6
Num. H-bond donors	0
Molar Refractivity	118.46
TPSA	74.30 Å²
**Lipophilicity**	
Log $P_{o/w}$ (iLOGP)	3.43
Log $P_{o/w}$ (XLOGP3)	4.28
Log $P_{o/w}$ (WLOGP)	3.49
Log $P_{o/w}$ (MLOGP)	1.00
Log $P_{o/w}$ (SILICOS-IT)	4.03
Consensus Log $P_{o/w}$	3.25

Water Solubility	
Log *S* (ESOL)	-5.05
Solubility	3.75e-03 mg/ml ; 8.87e-06 mol/l
Class	Moderately soluble
Log *S* (Ali)	-5.55
Solubility	1.19e-03 mg/ml ; 2.80e-06 mol/l
Class	Moderately soluble
Log *S* (SILICOS-IT)	-6.52
Solubility	1.28e-04 mg/ml ; 3.02e-07 mol/l
Class	Poorly soluble
**Pharmacokinetics**	
GI absorption	High
BBB permeant	Yes
P-gp substrate	No
CYP1A2 inhibitor	No
CYP2C19 inhibitor	Yes
CYP2C9 inhibitor	Yes
CYP2D6 inhibitor	Yes
CYP3A4 inhibitor	Yes
Log K_p (skin permeation)	-5.84 cm/s
**Druglikeness**	
Lipinski	Yes; 0 violation
Ghose	Yes
Veber	Yes
Egan	Yes
Muegge	Yes
Bioavailability Score	0.56
**Medicinal Chemistry**	
PAINS	1 alert: quinone_A
Brenk	0 alert
Leadlikeness	No; 2 violations: MW>350, XLOGP3>3.5
Synthetic accessibility	3.92

Molecule 2

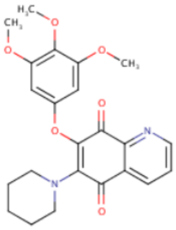

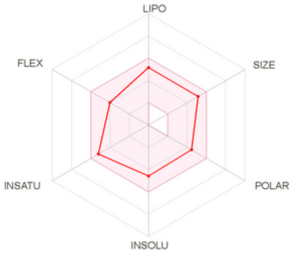

SMILES COc1cc(OC2=C(N3CCCCC3)C(=O)c3c(C2=O)nccc3)cc(c1OC)OC

Physicochemical Properties	
Formula	C23H24N2O6
Molecular weight	424.45 g/mol
Num. heavy atoms	31
Num. arom. heavy atoms	12
Fraction Csp3	0.35
Num. rotatable bonds	6
Num. H-bond acceptors	7
Num. H-bond donors	0
Molar Refractivity	116.26
TPSA	87.19 Å²
**Lipophilicity**	
Log $P_{o/w}$ (iLOGP)	3.24
Log $P_{o/w}$ (XLOGP3)	3.54
Log $P_{o/w}$ (WLOGP)	2.88
Log $P_{o/w}$ (MLOGP)	0.00
Log $P_{o/w}$ (SILICOS-IT)	3.46
Consensus Log $P_{o/w}$	2.62

Water Solubility	
Log *S* (ESOL)	-4.59
Solubility	1.09e-02 mg/ml ; 2.56e-05 mol/l
Class	Moderately soluble
Log *S* (Ali)	-5.06
Solubility	3.73e-03 mg/ml ; 8.80e-06 mol/l
Class	Moderately soluble
Log *S* (SILICOS-IT)	-6.15
Solubility	3.03e-04 mg/ml ; 7.13e-07 mol/l
Class	Poorly soluble
**Pharmacokinetics**	
GI absorption	High
BBB permeant	No
P-gp substrate	No
CYP1A2 inhibitor	No
CYP2C19 inhibitor	Yes
CYP2C9 inhibitor	Yes
CYP2D6 inhibitor	No
CYP3A4 inhibitor	Yes
Log K_p (skin permeation)	-6.38 cm/s
**Druglikeness**	
Lipinski	Yes; 0 violation
Ghose	Yes
Veber	Yes
Egan	Yes
Muegge	Yes
Bioavailability Score	0.56
**Medicinal Chemistry**	
PAINS	1 alert: quinone_A
Brenk	0 alert
Leadlikeness	No; 2 violations: MW>350, XLOGP3>3.5
Synthetic accessibility	3.85

Molecule 3

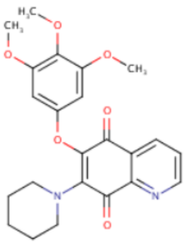

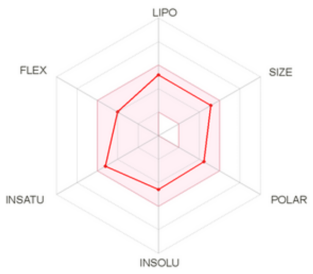

SMILES COc1cc(OC2=C(N3CCCCC3)C(=O)c3c(C2=O)cccn3)cc(c1OC)OC

Physicochemical Properties	
Formula	C23H24N2O6
Molecular weight	424.45 g/mol
Num. heavy atoms	31
Num. arom. heavy atoms	12
Fraction Csp3	0.35
Num. rotatable bonds	6
Num. H-bond acceptors	7
Num. H-bond donors	0
Molar Refractivity	116.26
TPSA	87.19 Å²

Lipophilicity	
Log $P_{o/w}$ (iLOGP)	3.23
Log $P_{o/w}$ (XLOGP3)	3.54
Log $P_{o/w}$ (WLOGP)	2.88
Log $P_{o/w}$ (MLOGP)	0.00
Log $P_{o/w}$ (SILICOS-IT)	3.46
Consensus Log $P_{o/w}$	2.62

Water Solubility	
Log S (ESOL)	-4.59
Solubility	1.09e-02 mg/ml ; 2.56e-05 mol/l
Class	Moderately soluble
Log S (Ali)	-5.06
Solubility	3.73e-03 mg/ml ; 8.80e-06 mol/l
Class	Moderately soluble
Log S (SILICOS-IT)	-6.15
Solubility	3.03e-04 mg/ml ; 7.13e-07 mol/l
Class	Poorly soluble

Pharmacokinetics	
GI absorption	High
BBB permeant	No
P-gp substrate	No
CYP1A2 inhibitor	No
CYP2C19 inhibitor	Yes
CYP2C9 inhibitor	Yes
CYP2D6 inhibitor	No
CYP3A4 inhibitor	Yes
Log K_p (skin permeation)	-6.38 cm/s

Druglikeness	
Lipinski	Yes; 0 violation
Ghose	Yes
Veber	Yes
Egan	Yes
Muegge	Yes
Bioavailability Score	0.56

Medicinal Chemistry	
PAINS	1 alert: quinone_A
Brenk	0 alert
Leadlikeness	No; 2 violations: MW>350, XLOGP3>3.5
Synthetic accessibility	3.82

Molecule 4

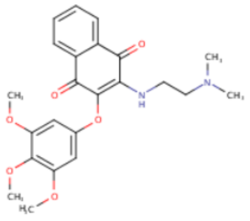

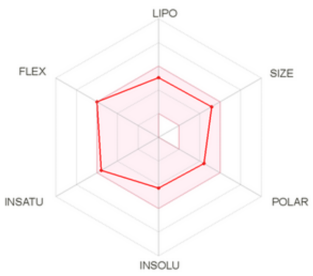

SMILES COc1cc(OC2=C(NCCN(C)C)C(=O)c3c(C2=O)cccc3)cc(c1OC)OC

Physicochemical Properties	
Formula	C23H26N2O6
Molecular weight	426.46 g/mol
Num. heavy atoms	31
Num. arom. heavy atoms	12
Fraction Csp3	0.30
Num. rotatable bonds	9
Num. H-bond acceptors	7
Num. H-bond donors	1
Molar Refractivity	114.66
TPSA	86.33 Å²

Lipophilicity	
Log $P_{o/w}$ (iLOGP)	3.75
Log $P_{o/w}$ (XLOGP3)	3.33
Log $P_{o/w}$ (WLOGP)	2.53
Log $P_{o/w}$ (MLOGP)	0.00
Log $P_{o/w}$ (SILICOS-IT)	3.19
Consensus Log $P_{o/w}$	2.56

Water Solubility	
Log S (ESOL)	-4.27
Solubility	2.27e-02 mg/ml ; 5.32e-05 mol/l
Class	Moderately soluble
Log S (Ali)	-4.82
Solubility	6.46e-03 mg/ml ; 1.51e-05 mol/l
Class	Moderately soluble
Log S (SILICOS-IT)	-6.76
Solubility	7.33e-05 mg/ml ; 1.72e-07 mol/l
Class	Poorly soluble

Pharmacokinetics	
GI absorption	High
BBB permeant	No
P-gp substrate	No
CYP1A2 inhibitor	Yes
CYP2C19 inhibitor	Yes
CYP2C9 inhibitor	Yes
CYP2D6 inhibitor	Yes
CYP3A4 inhibitor	Yes
Log K_p (skin permeation)	-6.54 cm/s

Druglikeness	
Lipinski	Yes; 0 violation
Ghose	Yes
Veber	Yes
Egan	Yes
Muegge	Yes
Bioavailability Score	0.56

Medicinal Chemistry	
PAINS	1 alert: quinone_A
Brenk	0 alert
Leadlikeness	No; 2 violations: MW>350, Rotors>7
Synthetic accessibility	4.02

Molecule 5

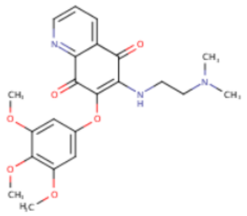

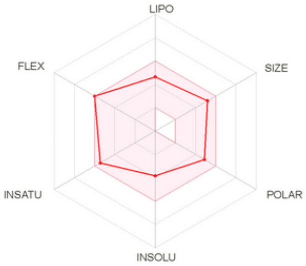

SMILES COc1cc(OC2=C(NCCN(C)C)C(=O)c3c(C2=O)nccc3)cc(c1OC)OC

Physicochemical Properties	
Formula	C22H25N3O6
Molecular weight	427.45 g/mol
Num. heavy atoms	31
Num. arom. heavy atoms	12
Fraction Csp3	0.32
Num. rotatable bonds	9
Num. H-bond acceptors	8
Num. H-bond donors	1
Molar Refractivity	112.45
TPSA	99.22 Å²

Lipophilicity	
Log $P_{o/w}$ (iLOGP)	3.06
Log $P_{o/w}$ (XLOGP3)	2.60
Log $P_{o/w}$ (WLOGP)	1.93
Log $P_{o/w}$ (MLOGP)	-0.99
Log $P_{o/w}$ (SILICOS-IT)	2.62
Consensus Log $P_{o/w}$	1.84

Water Solubility	
Log *S* (ESOL)	-3.82
Solubility	6.46e-02 mg/ml ; 1.51e-04 mol/l
Class	Soluble
Log *S* (Ali)	-4.33
Solubility	1.99e-02 mg/ml ; 4.65e-05 mol/l
Class	Moderately soluble
Log *S* (SILICOS-IT)	-6.39
Solubility	1.73e-04 mg/ml ; 4.06e-07 mol/l
Class	Poorly soluble

Pharmacokinetics	
GI absorption	High
BBB permeant	No
P-gp substrate	Yes
CYP1A2 inhibitor	Yes
CYP2C19 inhibitor	Yes
CYP2C9 inhibitor	Yes
CYP2D6 inhibitor	No
CYP3A4 inhibitor	Yes
Log K_p (skin permeation)	-7.06 cm/s

Druglikeness	
Lipinski	Yes; 0 violation
Ghose	Yes
Veber	Yes
Egan	Yes
Muegge	Yes
Bioavailability Score	0.56

Medicinal Chemistry	
PAINS	1 alert: quinone_A
Brenk	0 alert
Leadlikeness	No; 2 violations: MW>350, Rotors>7
Synthetic accessibility	3.94

Molecule 6

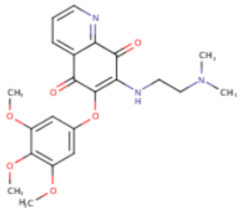

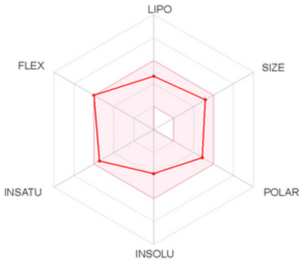

SMILES COc1cc(OC2=C(NCCN(C)C)C(=O)c3c(C2=O)cccn3)cc(c1OC)OC

Physicochemical Properties	
Formula	C22H25N3O6
Molecular weight	427.45 g/mol
Num. heavy atoms	31
Num. arom. heavy atoms	12
Fraction Csp3	0.32
Num. rotatable bonds	9
Num. H-bond acceptors	8
Num. H-bond donors	1
Molar Refractivity	112.45
TPSA	99.22 Å²

Lipophilicity	
Log $P_{o/w}$ (iLOGP)	2.97
Log $P_{o/w}$ (XLOGP3)	2.60
Log $P_{o/w}$ (WLOGP)	1.93
Log $P_{o/w}$ (MLOGP)	-0.99
Log $P_{o/w}$ (SILICOS-IT)	2.62
Consensus Log $P_{o/w}$	1.83

Water Solubility	
Log *S* (ESOL)	-3.82
Solubility	6.46e-02 mg/ml ; 1.51e-04 mol/l
Class	Soluble
Log *S* (Ali)	-4.33
Solubility	1.99e-02 mg/ml ; 4.65e-05 mol/l
Class	Moderately soluble
Log *S* (SILICOS-IT)	-6.39
Solubility	1.73e-04 mg/ml ; 4.06e-07 mol/l
Class	Poorly soluble

Pharmacokinetics	
GI absorption	High
BBB permeant	No
P-gp substrate	Yes
CYP1A2 inhibitor	Yes
CYP2C19 inhibitor	Yes
CYP2C9 inhibitor	Yes
CYP2D6 inhibitor	No
CYP3A4 inhibitor	Yes
Log K_p (skin permeation)	-7.06 cm/s

Druglikeness	
Lipinski	Yes; 0 violation
Ghose	Yes
Veber	Yes
Egan	Yes
Muegge	Yes
Bioavailability Score	0.56

Medicinal Chemistry	
PAINS	1 alert: quinone_A
Brenk	0 alert
Leadlikeness	No; 2 violations: MW>350, Rotors>7
Synthetic accessibility	3.89

Molecule 7

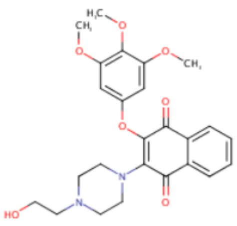

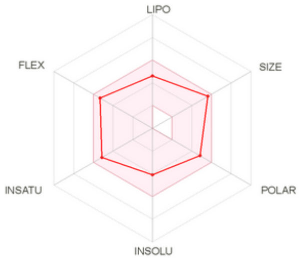

SMILES OCCN1CCN(CC1)C1=C(Oc2cc(OC)c(c(c2)OC)OC)C(=O)c2c(C1=O)cccc2

Physicochemical Properties	
Formula	C25H28N2O7
Molecular weight	468.50 g/mol
Num. heavy atoms	34
Num. arom. heavy atoms	12
Fraction Csp3	0.36
Num. rotatable bonds	8
Num. H-bond acceptors	8
Num. H-bond donors	1
Molar Refractivity	131.24
TPSA	97.77 Å²

Lipophilicity	
Log $P_{o/w}$ (iLOGP)	3.67
Log $P_{o/w}$ (XLOGP3)	2.56
Log $P_{o/w}$ (WLOGP)	1.23
Log $P_{o/w}$ (MLOGP)	-0.36
Log $P_{o/w}$ (SILICOS-IT)	2.82
Consensus Log $P_{o/w}$	1.98

Water Solubility	
Log *S* (ESOL)	-4.09
Solubility	3.80e-02 mg/ml ; 8.12e-05 mol/l
Class	Moderately soluble
Log *S* (Ali)	-4.26
Solubility	2.57e-02 mg/ml ; 5.48e-05 mol/l
Class	Moderately soluble
Log *S* (SILICOS-IT)	-5.77
Solubility	7.99e-04 mg/ml ; 1.71e-06 mol/l
Class	Moderately soluble

Pharmacokinetics	
GI absorption	High
BBB permeant	No
P-gp substrate	Yes
CYP1A2 inhibitor	No
CYP2C19 inhibitor	Yes
CYP2C9 inhibitor	Yes
CYP2D6 inhibitor	No
CYP3A4 inhibitor	Yes
Log K_p (skin permeation)	-7.34 cm/s

Druglikeness	
Lipinski	Yes; 0 violation
Ghose	No; 1 violation: MR>130
Veber	Yes
Egan	Yes
Muegge	Yes
Bioavailability Score	0.56

Medicinal Chemistry	
PAINS	1 alert: quinone_A
Brenk	0 alert
Leadlikeness	No; 2 violations: MW>350, Rotors>7
Synthetic accessibility	4.20

Reference Molecules

Molecule 8

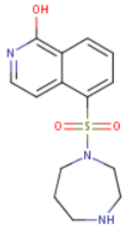

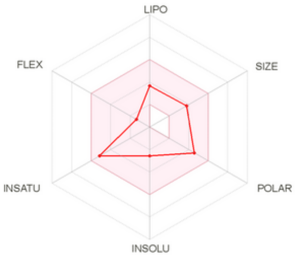

SMILES Oc1nccc2c1cccc2S(=O)(=O)N1CCNCCC1

Physicochemical Properties	
Formula	C14H17N3O3S
Molecular weight	307.37 g/mol
Num. heavy atoms	21
Num. arom. heavy atoms	10
Fraction Csp3	0.36
Num. rotatable bonds	2
Num. H-bond acceptors	6
Num. H-bond donors	2
Molar Refractivity	87.50
TPSA	90.91 Å²

Lipophilicity	
Log $P_{o/w}$ (iLOGP)	1.86
Log $P_{o/w}$ (XLOGP3)	0.93
Log $P_{o/w}$ (WLOGP)	1.24
Log $P_{o/w}$ (MLOGP)	0.55
Log $P_{o/w}$ (SILICOS-IT)	0.59
Consensus Log $P_{o/w}$	1.04

Water Solubility	
Log *S* (ESOL)	-2.55
Solubility	8.62e-01 mg/ml ; 2.81e-03 mol/l
Class	Soluble
Log *S* (Ali)	-2.43
Solubility	1.15e+00 mg/ml ; 3.76e-03 mol/l
Class	Soluble
Log *S* (SILICOS-IT)	-3.81
Solubility	4.73e-02 mg/ml ; 1.54e-04 mol/l
Class	Soluble

Pharmacokinetics	
GI absorption	High
BBB permeant	No
P-gp substrate	Yes
CYP1A2 inhibitor	No
CYP2C19 inhibitor	No
CYP2C9 inhibitor	No
CYP2D6 inhibitor	No
CYP3A4 inhibitor	No
Log K_p (skin permeation)	-7.51 cm/s

Druglikeness	
Lipinski	Yes; 0 violation
Ghose	Yes
Veber	Yes
Egan	Yes
Muegge	Yes
Bioavailability Score	0.55

Medicinal Chemistry	
PAINS	0 alert
Brenk	0 alert
Leadlikeness	Yes
Synthetic accessibility	2.71

Molecule 9

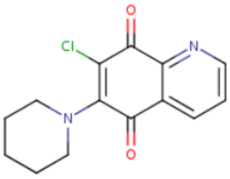

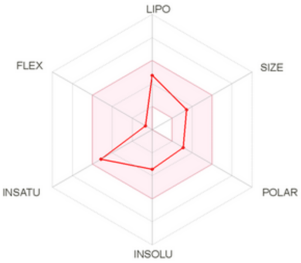

SMILES ClC1=C(N2CCCCC2)C(=O)c2c(C1=O)nccc2

Physicochemical Properties	
Formula	C14H13ClN2O2
Molecular weight	276.72 g/mol
Num. heavy atoms	19
Num. arom. heavy atoms	6
Fraction Csp3	0.36
Num. rotatable bonds	1
Num. H-bond acceptors	3
Num. H-bond donors	0
Molar Refractivity	75.57
TPSA	50.27 Å²

Lipophilicity	
Log $P_{o/w}$ (iLOGP)	1.88
Log $P_{o/w}$ (XLOGP3)	2.73
Log $P_{o/w}$ (WLOGP)	2.02
Log $P_{o/w}$ (MLOGP)	0.55
Log $P_{o/w}$ (SILICOS-IT)	2.88
Consensus Log $P_{o/w}$	2.01

Water Solubility	
Log *S* (ESOL)	-3.44
Solubility	9.97e-02 mg/ml ; 3.60e-04 mol/l
Class	Soluble
Log *S* (Ali)	-3.44
Solubility	1.01e-01 mg/ml ; 3.63e-04 mol/l
Class	Soluble
Log *S* (SILICOS-IT)	-4.22
Solubility	1.66e-02 mg/ml ; 6.01e-05 mol/l
Class	Moderately soluble

Pharmacokinetics	
GI absorption	High
BBB permeant	Yes
P-gp substrate	No
CYP1A2 inhibitor	Yes
CYP2C19 inhibitor	Yes
CYP2C9 inhibitor	No
CYP2D6 inhibitor	No
CYP3A4 inhibitor	Yes
Log K_p (skin permeation)	-6.05 cm/s

Druglikeness	
Lipinski	Yes; 0 violation
Ghose	Yes
Veber	Yes
Egan	Yes
Muegge	Yes
Bioavailability Score	0.55

Medicinal Chemistry	
PAINS	2 alerts: ene_one_hal, quinone_A
Brenk	0 alert
Leadlikeness	Yes
Synthetic accessibility	2.94

Molecule 10

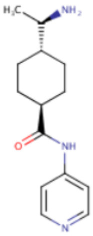

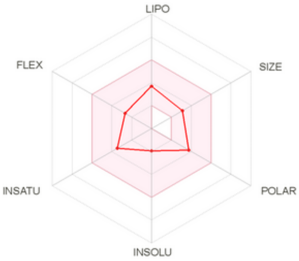

SMILES C[C@H]([C@@H]1CC[C@H](CC1)C(=O)Nc1ccncc1)N

Physicochemical Properties	
Formula	C14H21N3O
Molecular weight	247.34 g/mol
Num. heavy atoms	18
Num. arom. heavy atoms	6
Fraction Csp3	0.57
Num. rotatable bonds	4
Num. H-bond acceptors	3
Num. H-bond donors	2
Molar Refractivity	72.79
TPSA	68.01 Å²

Lipophilicity	
Log $P_{o/w}$ (iLOGP)	2.20
Log $P_{o/w}$ (XLOGP3)	0.95
Log $P_{o/w}$ (WLOGP)	1.98
Log $P_{o/w}$ (MLOGP)	1.13
Log $P_{o/w}$ (SILICOS-IT)	1.56
Consensus Log $P_{o/w}$	1.57

Water Solubility	
Log *S* (ESOL)	-1.95
Solubility	2.75e+00 mg/ml ; 1.11e-02 mol/l
Class	Very soluble
Log *S* (Ali)	-1.97
Solubility	2.68e+00 mg/ml ; 1.08e-02 mol/l
Class	Very soluble
Log *S* (SILICOS-IT)	-3.31
Solubility	1.20e-01 mg/ml ; 4.85e-04 mol/l
Class	Soluble

Pharmacokinetics	
GI absorption	High
BBB permeant	Yes
P-gp substrate	No
CYP1A2 inhibitor	No
CYP2C19 inhibitor	No
CYP2C9 inhibitor	No
CYP2D6 inhibitor	No
CYP3A4 inhibitor	No
Log K_p (skin permeation)	-7.13 cm/s

Druglikeness	
Lipinski	Yes; 0 violation
Ghose	Yes
Veber	Yes
Egan	Yes
Muegge	Yes
Bioavailability Score	0.55

Medicinal Chemistry	
PAINS	0 alert
Brenk	0 alert
Leadlikeness	No; 1 violation: MW<250
Synthetic accessibility	2.73

Molecule 11

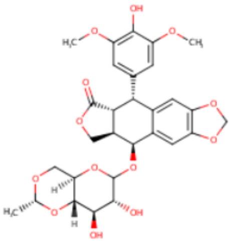

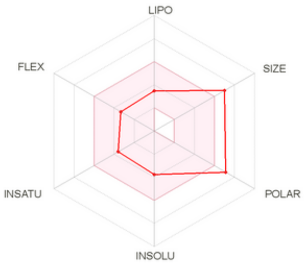

SMILES COc1cc(cc(c1O)OC)[C@H]1[C@H]2C(=O)OC[C@@H]2[C@@H](c2cc1OCOc1c2)OC1O[C@@H]2CO[C@H](O[C@H]2[C@@H]([C@H]1O)O)C

Physicochemical Properties	
Formula	C29H32O13
Molecular weight	588.56 g/mol
Num. heavy atoms	42
Num. arom. heavy atoms	12
Fraction Csp3	0.55
Num. rotatable bonds	5
Num. H-bond acceptors	13
Num. H-bond donors	3
Molar Refractivity	139.11
TPSA	160.83 Å²

Lipophilicity	
Log $P_{o/w}$ (iLOGP)	3.24
Log $P_{o/w}$ (XLOGP3)	0.60
Log $P_{o/w}$ (WLOGP)	1.01
Log $P_{o/w}$ (MLOGP)	-0.14
Log $P_{o/w}$ (SILICOS-IT)	0.95
Consensus Log $P_{o/w}$	1.13

Water Solubility	
Log *S* (ESOL)	-3.75
Solubility	1.05e-01 mg/ml ; 1.78e-04 mol/l
Class	Soluble
Log *S* (Ali)	-3.55
Solubility	1.65e-01 mg/ml ; 2.81e-04 mol/l
Class	Soluble
Log *S* (SILICOS-IT)	-3.18
Solubility	3.85e-01 mg/ml ; 6.55e-04 mol/l
Class	Soluble

Pharmacokinetics	
GI absorption	Low
BBB permeant	No
P-gp substrate	Yes
CYP1A2 inhibitor	No
CYP2C19 inhibitor	No
CYP2C9 inhibitor	No
CYP2D6 inhibitor	Yes
CYP3A4 inhibitor	No
Log K_p (skin permeation)	-9.46 cm/s

Druglikeness	
Lipinski	No; 2 violations: MW>500, NorO>10
Ghose	No; 3 violations: MW>480, MR>130, #atoms>70
Veber	No; 1 violation: TPSA>140
Egan	No; 1 violation: TPSA>131.6
Muegge	No; 2 violations: TPSA>150, H-acc>10
Bioavailability Score	0.17

Medicinal Chemistry	
PAINS	0 alert
Brenk	0 alert
Leadlikeness	No; 1 violation: MW>350
Synthetic accessibility	6.27

Molecule 12

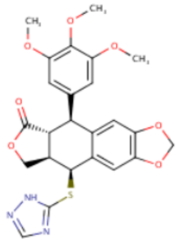

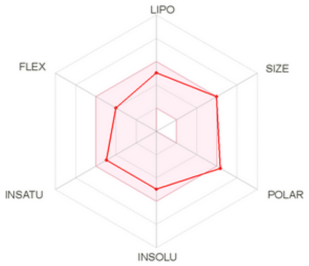

SMILES COc1c(OC)cc(cc1OC)[C@@H]1[C@H]2C(=O)OC[C@@H]2[C@@H](c2c1cc1OCOc1c2)Sc1[nH]ncn1

Physicochemical Properties	
Formula	C24H23N3O7S
Molecular weight	497.52 g/mol
Num. heavy atoms	35
Num. arom. heavy atoms	17
Fraction Csp3	0.38
Num. rotatable bonds	6
Num. H-bond acceptors	9
Num. H-bond donors	1
Molar Refractivity	123.87
TPSA	139.32 Å²

Lipophilicity	
Log $P_{o/w}$ (iLOGP)	2.78
Log $P_{o/w}$ (XLOGP3)	3.30
Log $P_{o/w}$ (WLOGP)	3.00
Log $P_{o/w}$ (MLOGP)	1.60
Log $P_{o/w}$ (SILICOS-IT)	3.41
Consensus Log $P_{o/w}$	2.82

Water Solubility	
Log *S* (ESOL)	-4.97
Solubility	5.37e-03 mg/ml ; 1.08e-05 mol/l
Class	Moderately soluble
Log *S* (Ali)	-5.90
Solubility	6.24e-04 mg/ml ; 1.26e-06 mol/l
Class	Moderately soluble
Log *S* (SILICOS-IT)	-6.34
Solubility	2.30e-04 mg/ml ; 4.61e-07 mol/l
Class	Poorly soluble

Pharmacokinetics	
GI absorption	Low
BBB permeant	No
P-gp substrate	No
CYP1A2 inhibitor	No
CYP2C19 inhibitor	No
CYP2C9 inhibitor	Yes
CYP2D6 inhibitor	Yes
CYP3A4 inhibitor	Yes
Log K_p (skin permeation)	-6.99 cm/s

Druglikeness	
Lipinski	Yes; 0 violation
Ghose	No; 1 violation: MW>480
Veber	Yes
Egan	No; 1 violation: TPSA>131.6
Muegge	Yes
Bioavailability Score	0.55

Medicinal Chemistry	
PAINS	0 alert
Brenk	0 alert
Leadlikeness	No; 1 violation: MW>350
Synthetic accessibility	5.13

Molecule 13

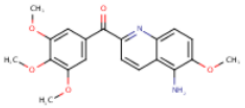

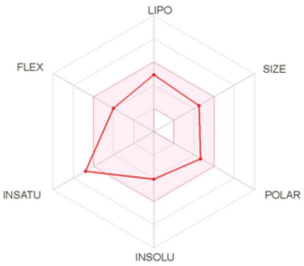

SMILES COc1cc(cc(c1OC)OC)C(=O)c1ccc2c(n1)ccc(c2N)OC

Physicochemical Properties	
Formula	C20H20N2O5
Molecular weight	368.38 g/mol
Num. heavy atoms	27
Num. arom. heavy atoms	16
Fraction Csp3	0.20
Num. rotatable bonds	6
Num. H-bond acceptors	6
Num. H-bond donors	1
Molar Refractivity	101.99
TPSA	92.90 Å²

Lipophilicity	
Log $P_{o/w}$ (iLOGP)	3.22
Log $P_{o/w}$ (XLOGP3)	3.07
Log $P_{o/w}$ (WLOGP)	3.09
Log $P_{o/w}$ (MLOGP)	0.63
Log $P_{o/w}$ (SILICOS-IT)	3.35
Consensus Log $P_{o/w}$	2.67

Water Solubility	
Log *S* (ESOL)	-4.10
Solubility	2.92e-02 mg/ml ; 7.93e-05 mol/l
Class	Moderately soluble
Log *S* (Ali)	-4.69
Solubility	7.56e-03 mg/ml ; 2.05e-05 mol/l
Class	Moderately soluble
Log *S* (SILICOS-IT)	-6.27
Solubility	2.00e-04 mg/ml ; 5.42e-07 mol/l
Class	Poorly soluble

Pharmacokinetics	
GI absorption	High
BBB permeant	No
P-gp substrate	No
CYP1A2 inhibitor	Yes
CYP2C19 inhibitor	Yes
CYP2C9 inhibitor	Yes
CYP2D6 inhibitor	Yes
CYP3A4 inhibitor	Yes
Log K_p (skin permeation)	-6.37 cm/s

Druglikeness	
Lipinski	Yes; 0 violation
Ghose	Yes
Veber	Yes
Egan	Yes
Muegge	Yes
Bioavailability Score	0.55

Medicinal Chemistry	
PAINS	0 alert
Brenk	1 alert: aniline
Leadlikeness	No; 1 violation: MW>350
Synthetic accessibility	2.83

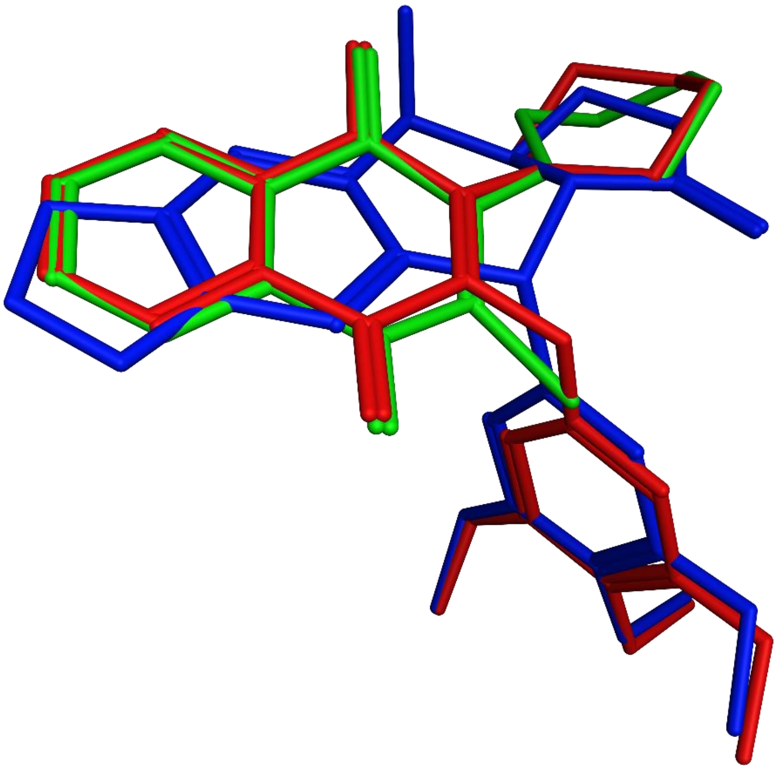

Figure S1. Overlapping of the energy minimized structures **1b** (in red), PT-262 (in green) and podophyllotoxin (in blue). Hydrogen atoms are omitted for clarity.

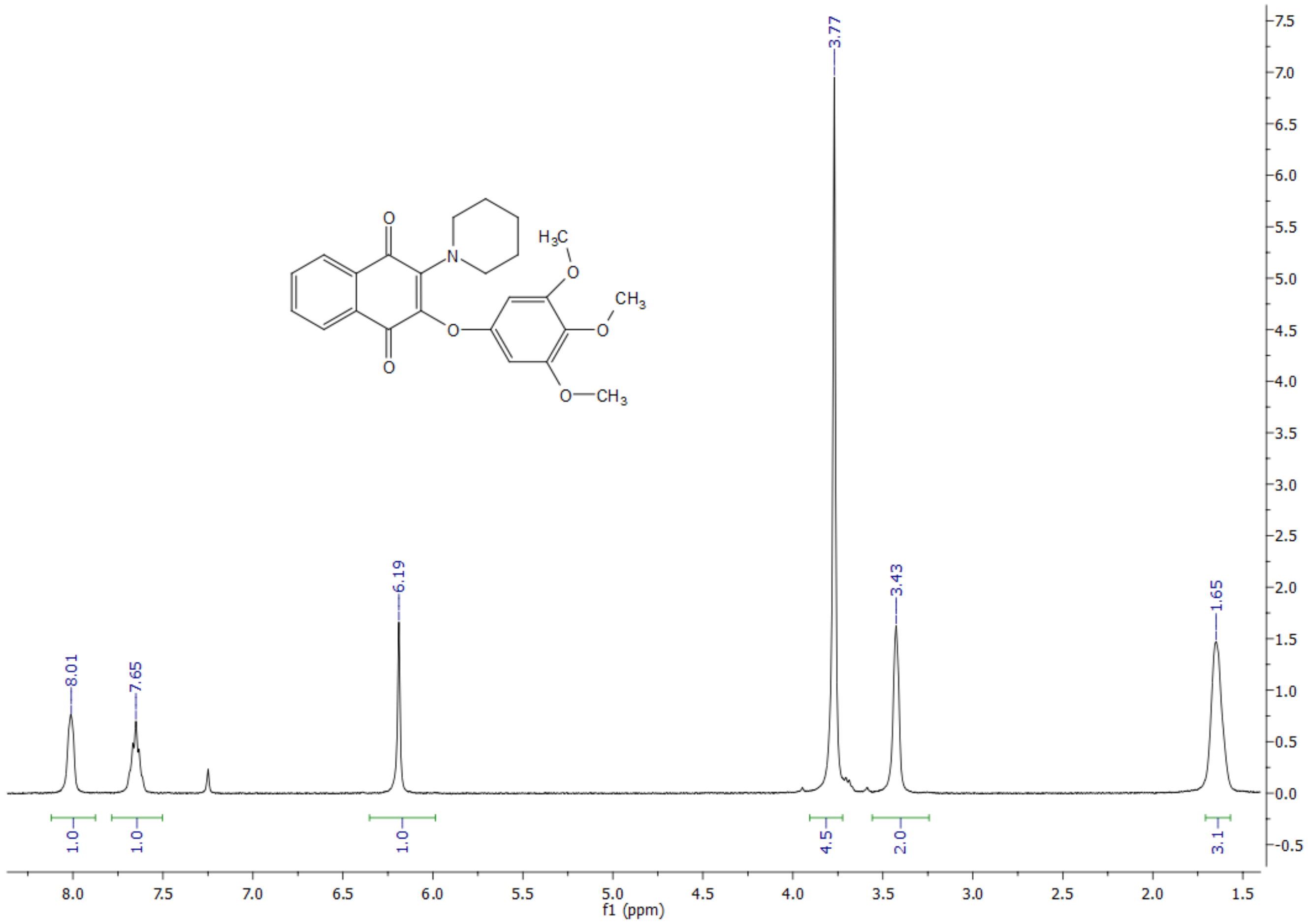

Figure S2. ^{1}HNMR spectrum (400MHz, $CDCl_3$) of compound **1a.**

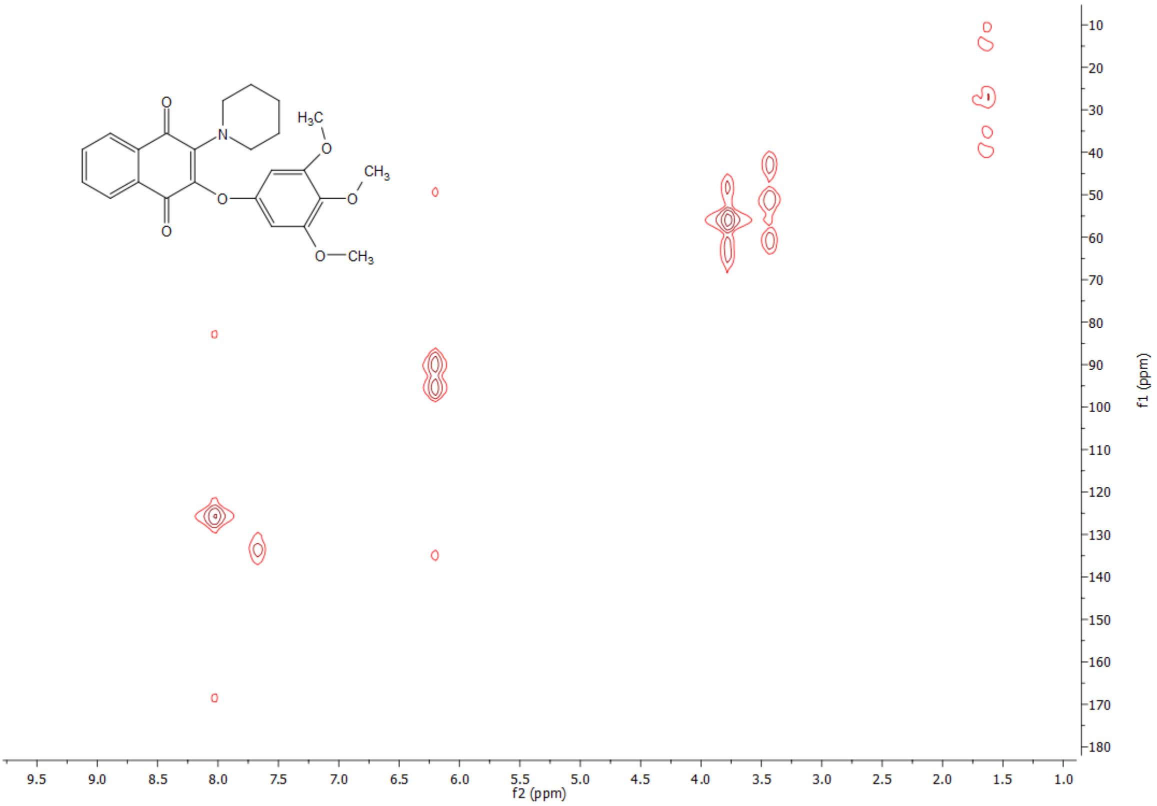

Figure S3. $^{1}H,^{13}C$ correlations by HSQC experiment (400MHz, $CDCl_3$) of **1a.**

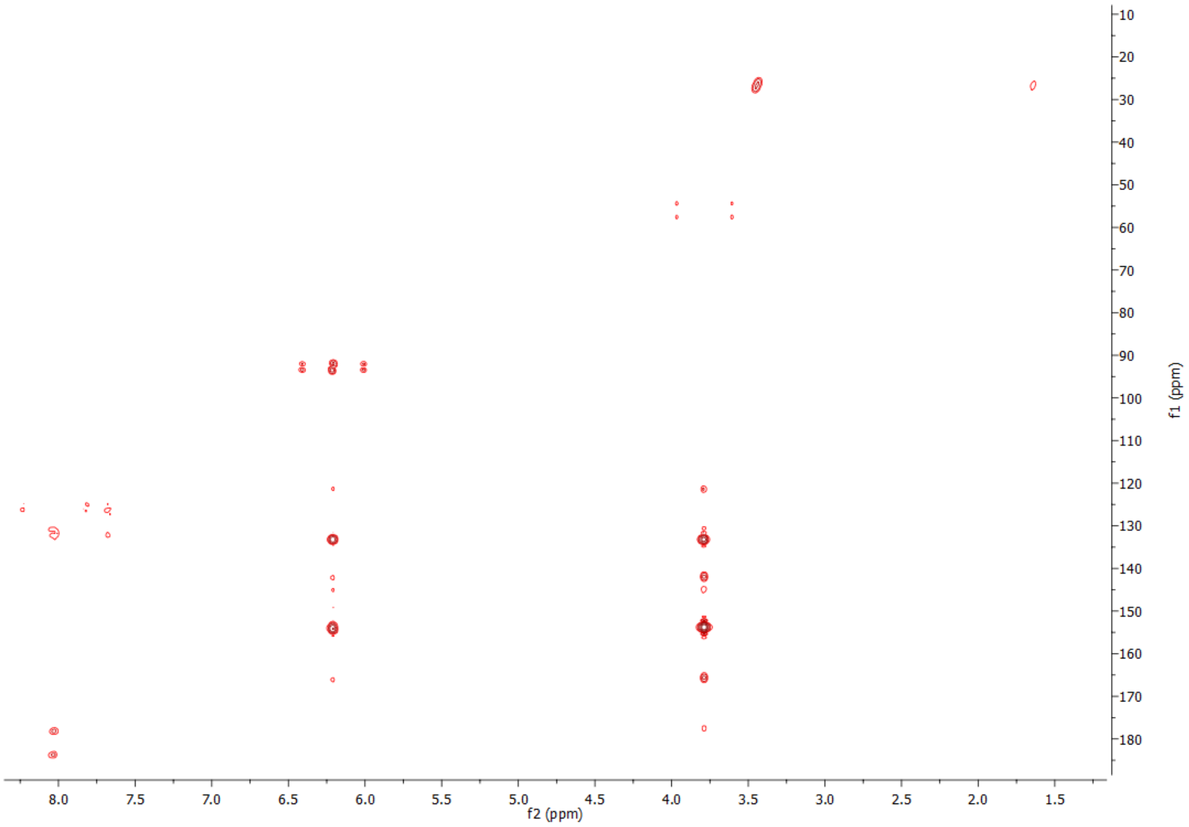

Figure S4. $^{1}H,^{13}C$ long range correlations by HMBC experiment (400MHz, $CDCl_3$) of **1a.**

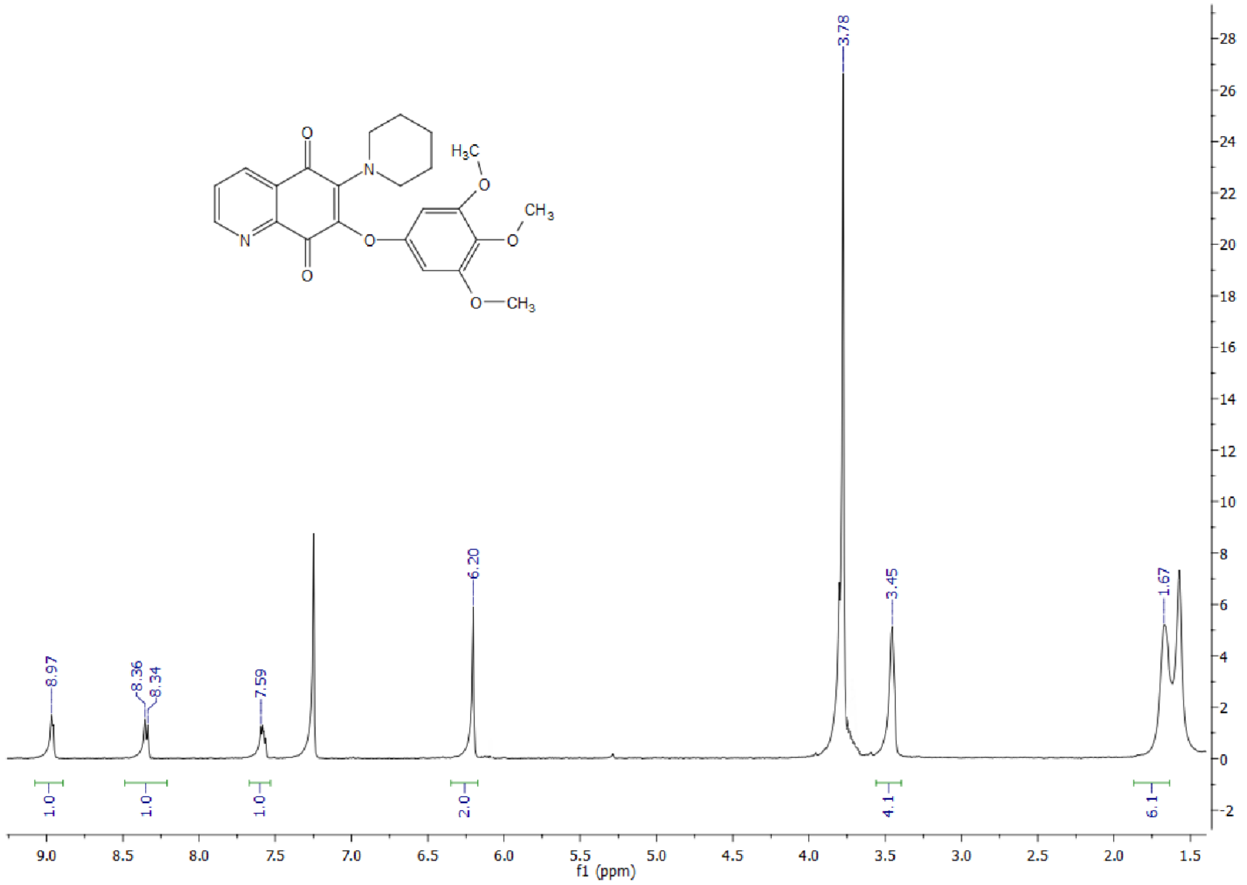

Figure S5. ^{1}HNMR spectrum (400MHz, $CDCl_3$) of compound **1b.**

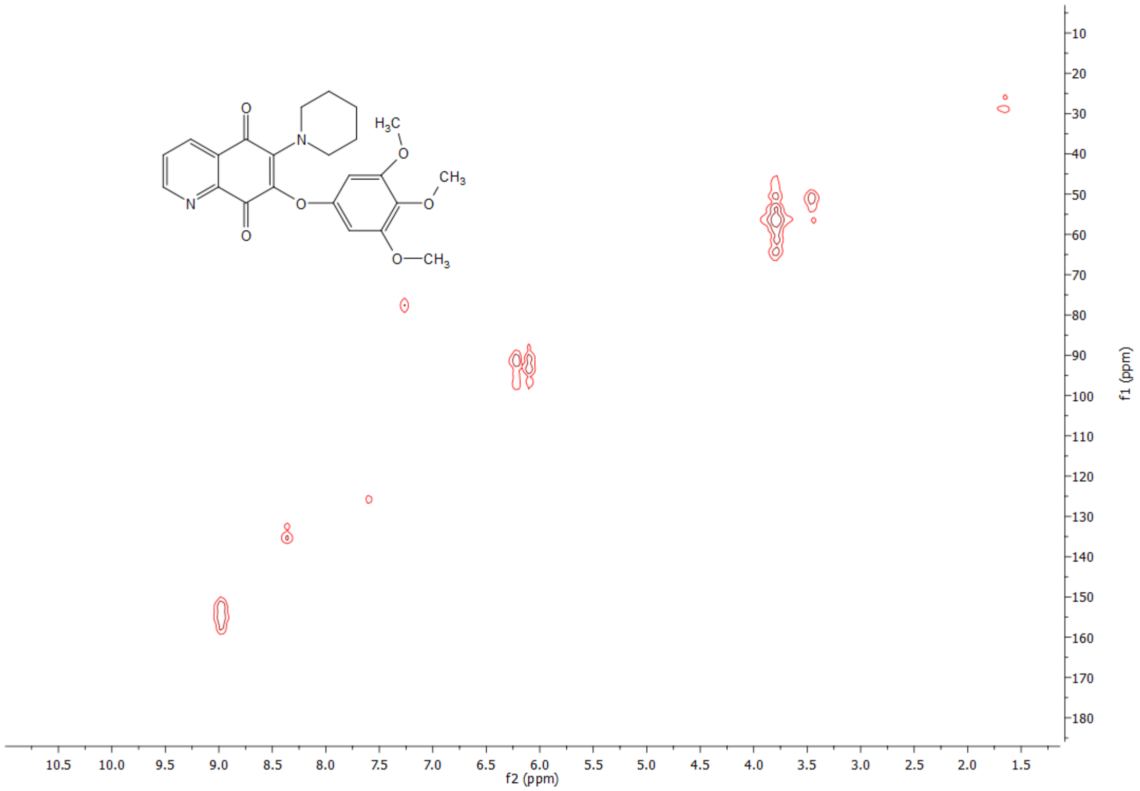

Figure S6. $^1H,^{13}C$ correlations by HSQC experiment (400MHz, CDCl3) of **1b.**

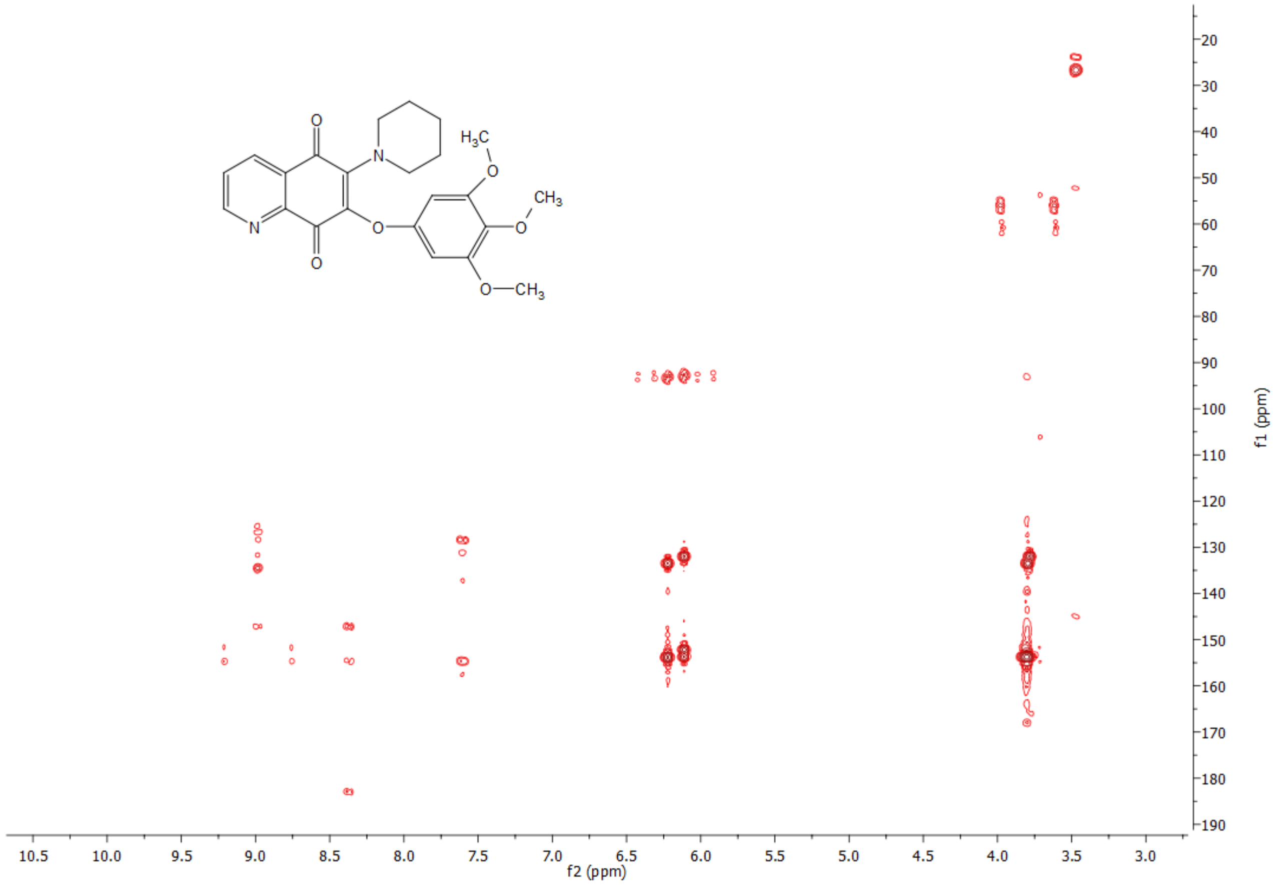

Figure S7. $^1H,^{13}C$ long range correlations by HMBC experiment (400MHz, CDCl3) of **1b.**

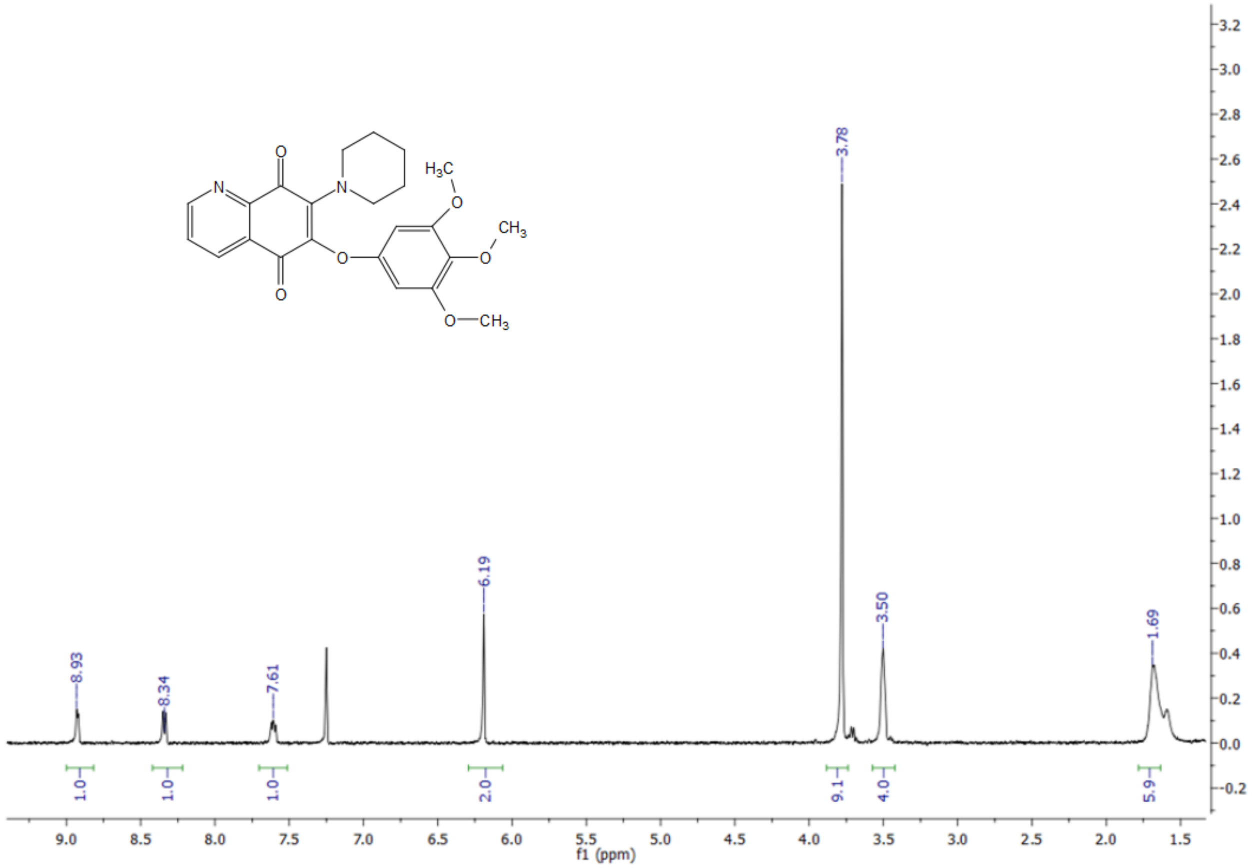

Figure S8. ^{1}HNMR spectrum (400MHz, $CDCl_3$) of compound **1c.**

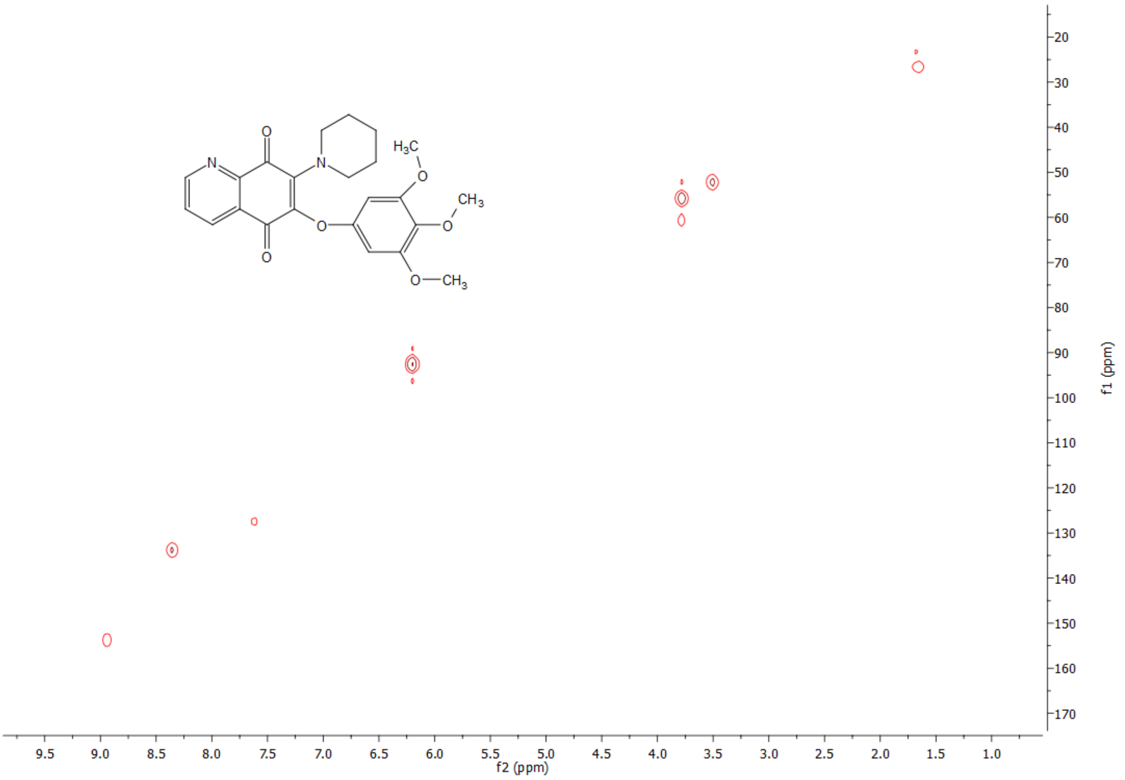

Figure S9. ^1H,^{13}C correlations by HSQC experiment (400MHz, $CDCl_3$) of **1c.**

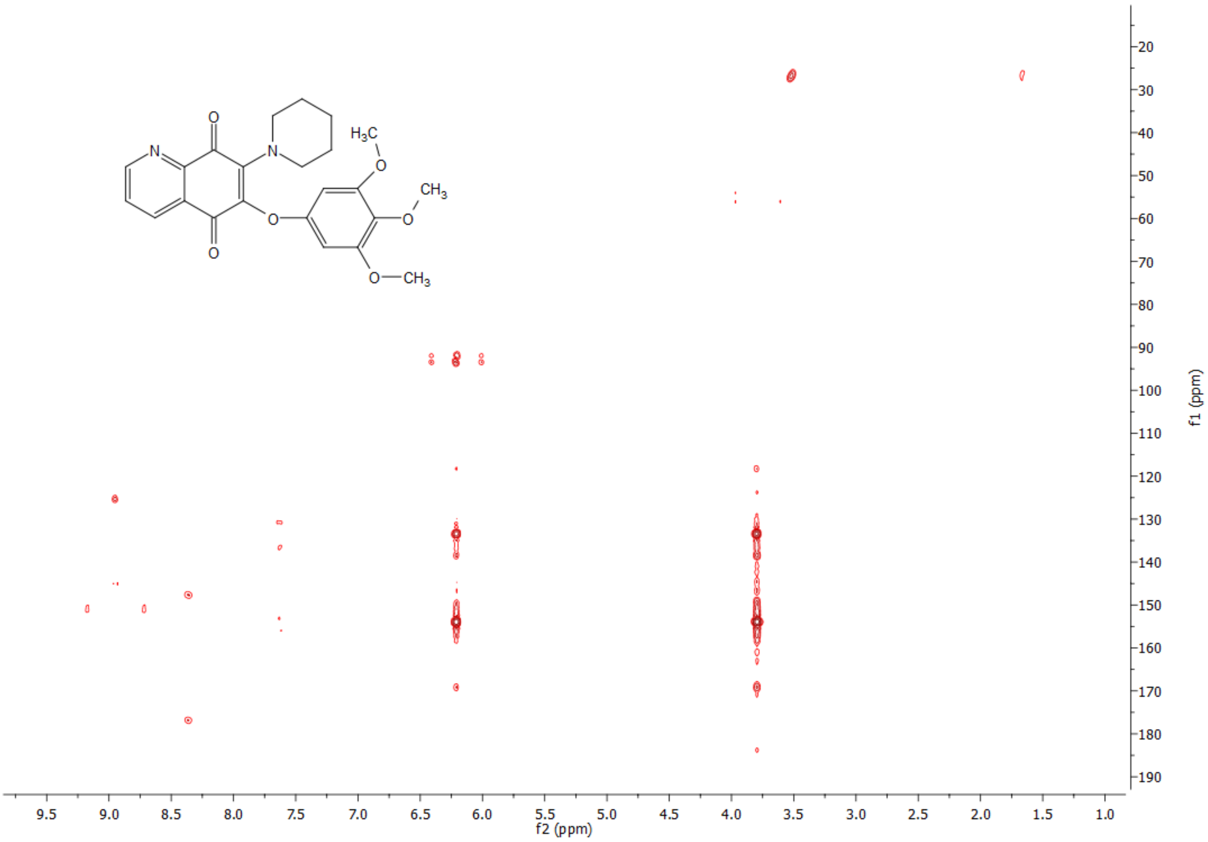

Figure S10. ^1H,^{13}C long range correlations by HMBC experiment (400MHz, $CDCl_3$) of **1c.**

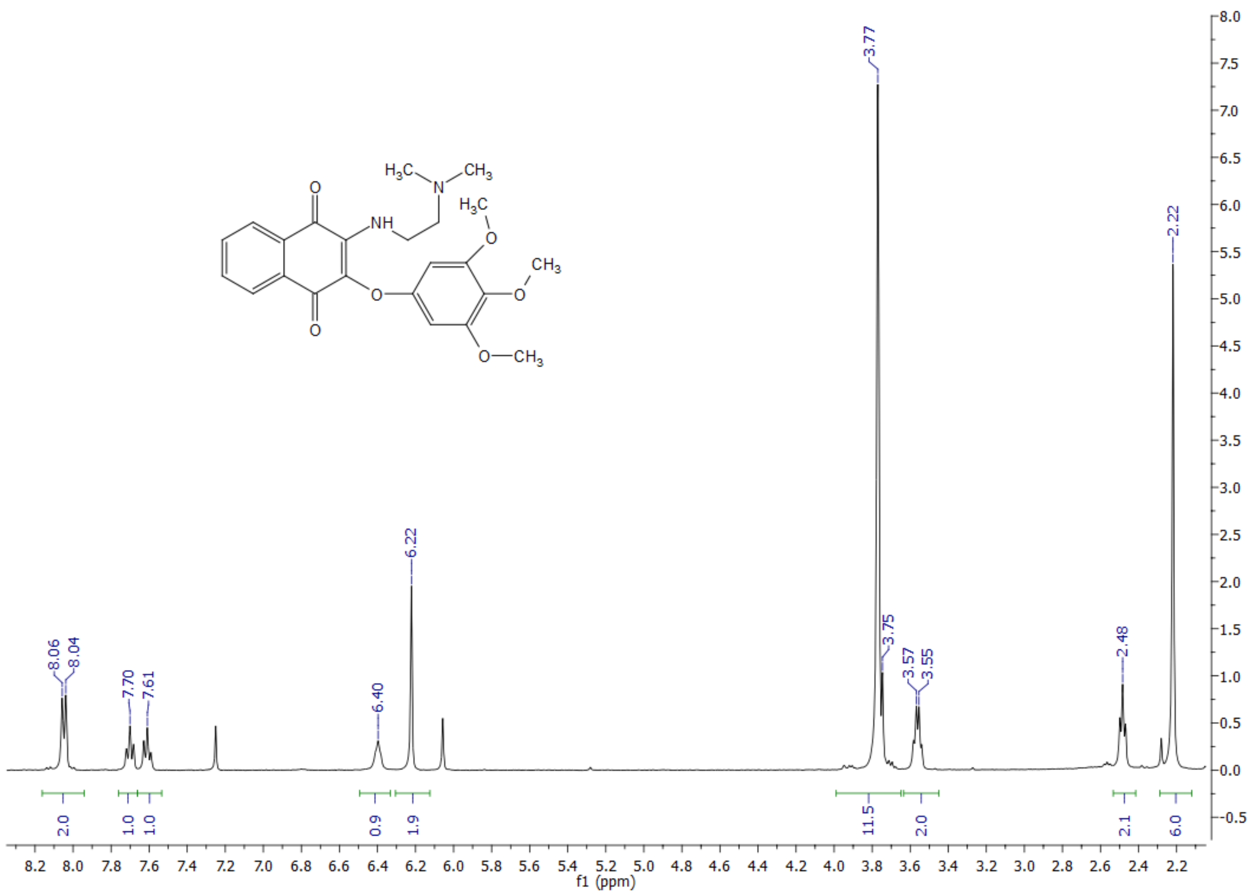

Figure S11. ^{1}HNMR spectrum (400MHz, $CDCl_3$) of compound **2a.**

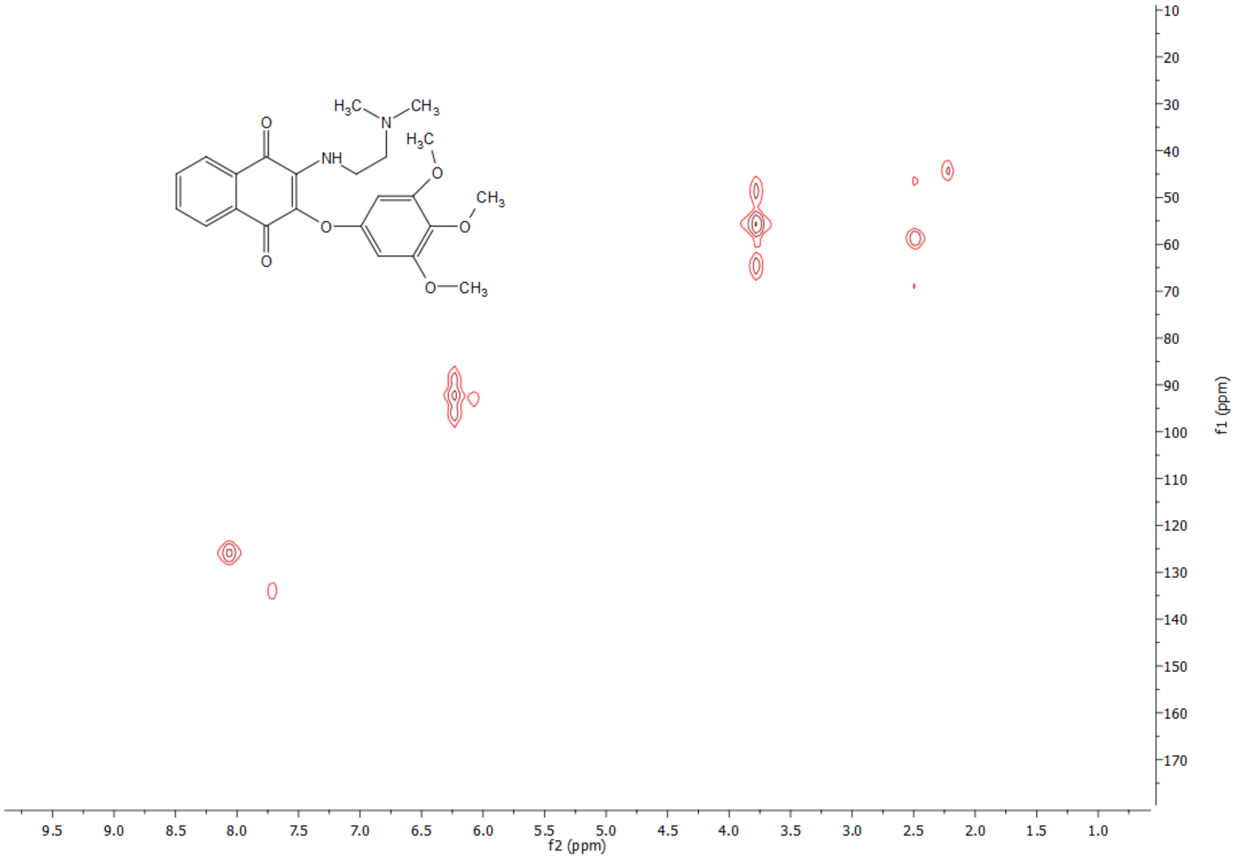

Figure S12. ^{1}H,^{13}C correlations by HSQC experiment (400MHz, $CDCl_3$) of **2a.**

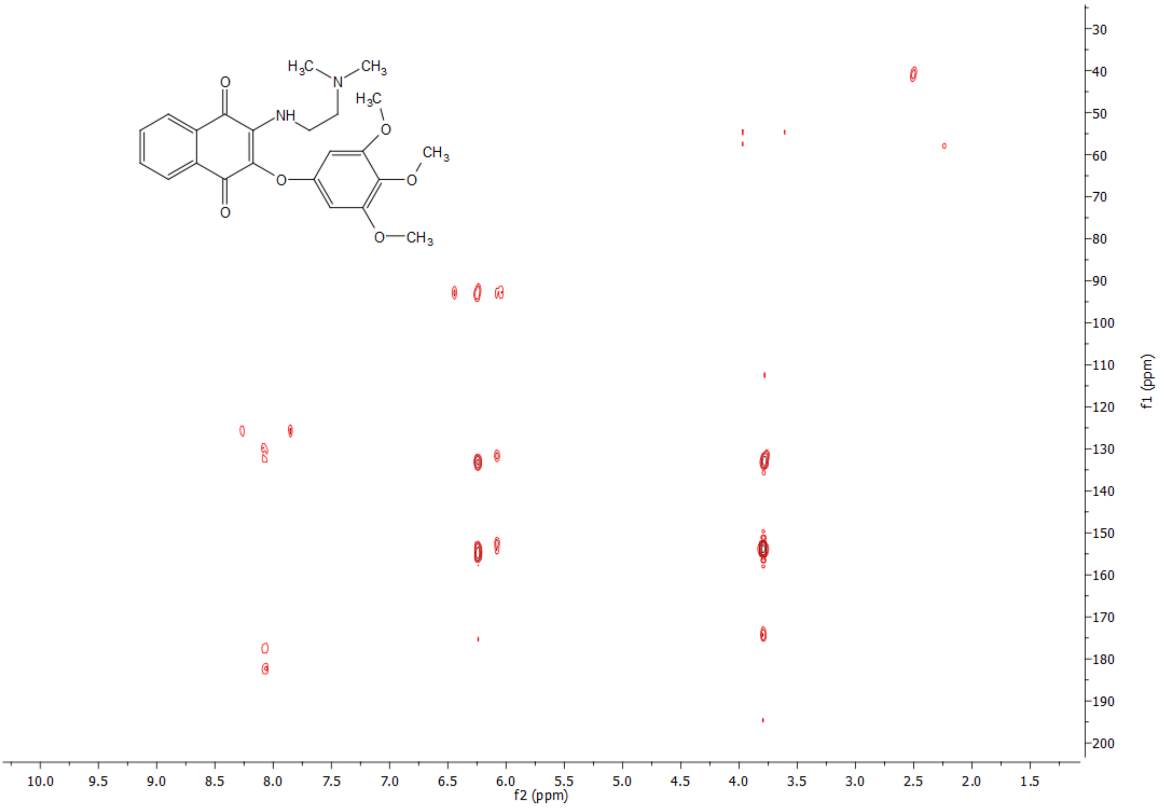

Figure S13. ^{1}H,^{13}C long range correlations by HMBC experiment (400MHz, $CDCl_3$) of **2a.**

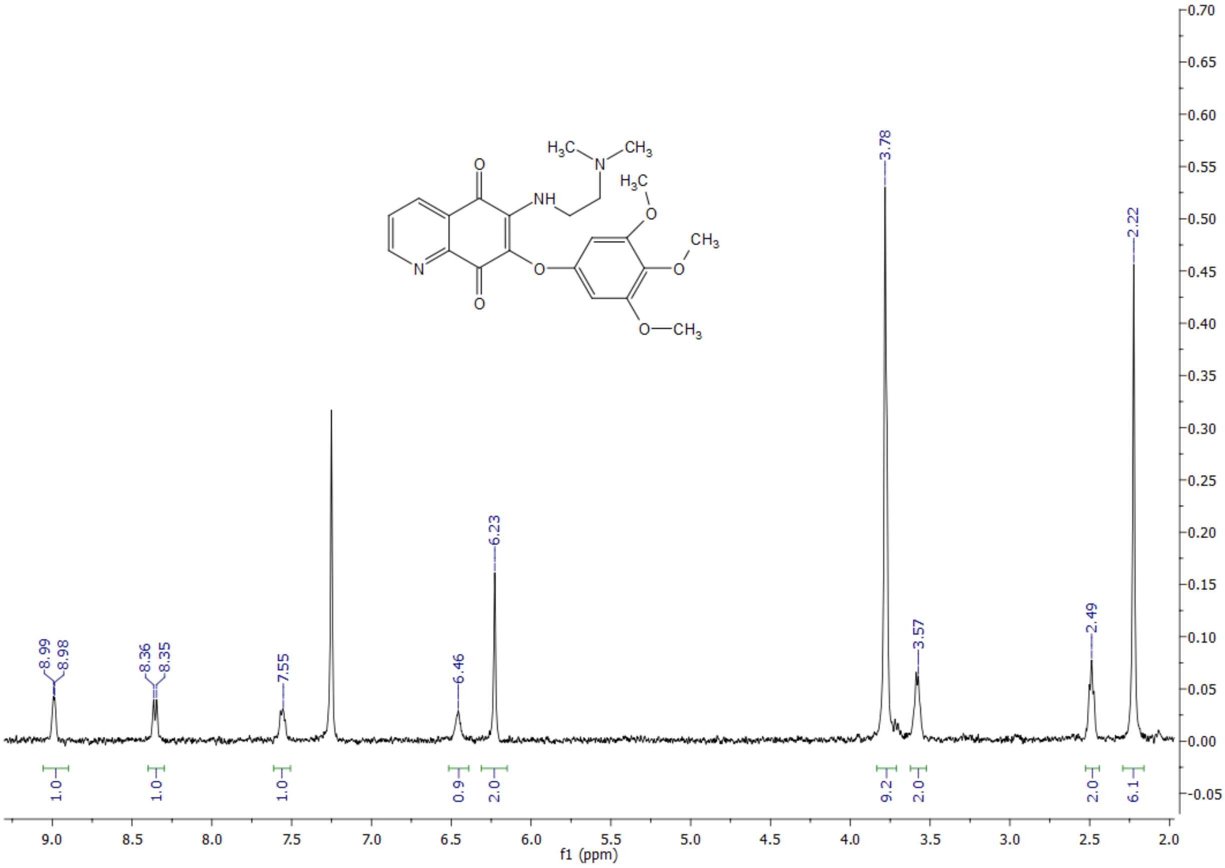

Figure S14. ^{1}HNMR spectrum (400MHz, $CDCl_3$) of compound **2b.**

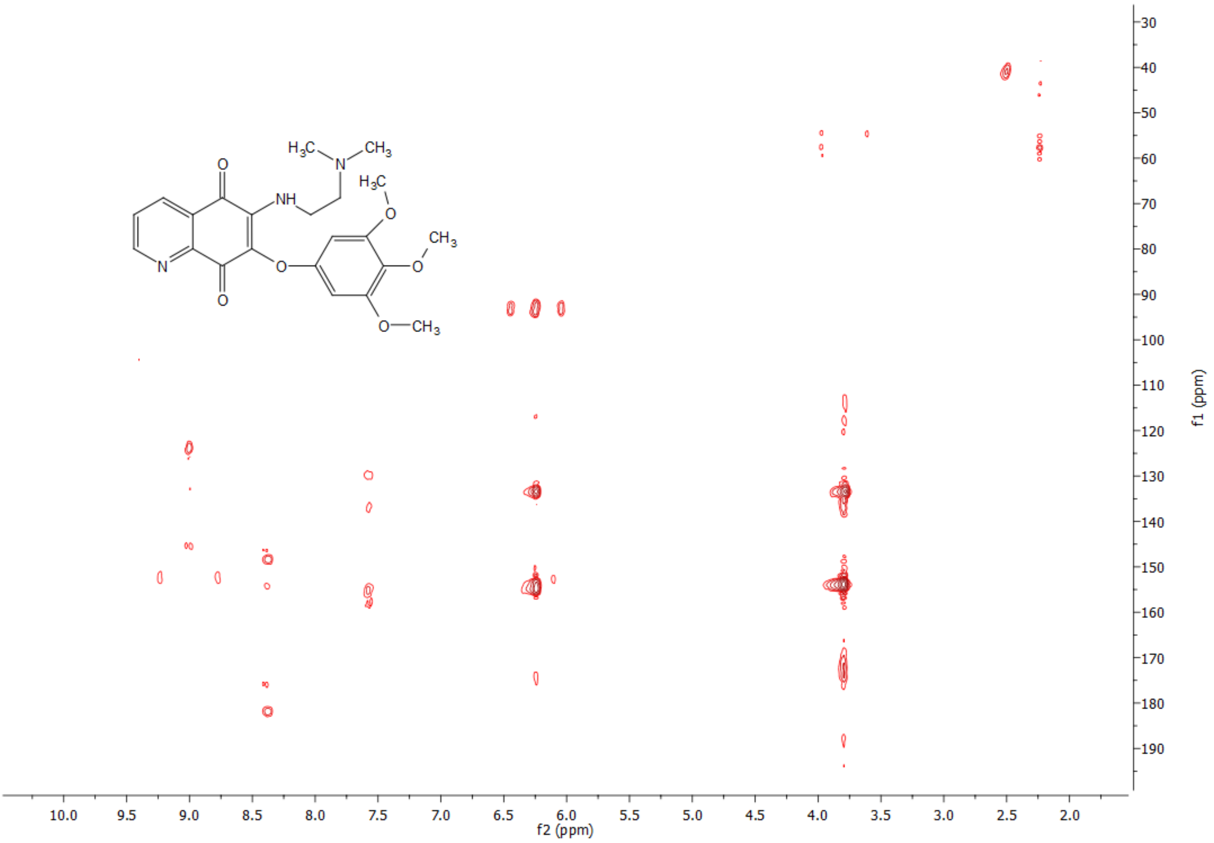

Figure S15. ^{1}H,^{13}C long range correlations by HMBC experiment (400MHz, $CDCl_3$) of **2b.**

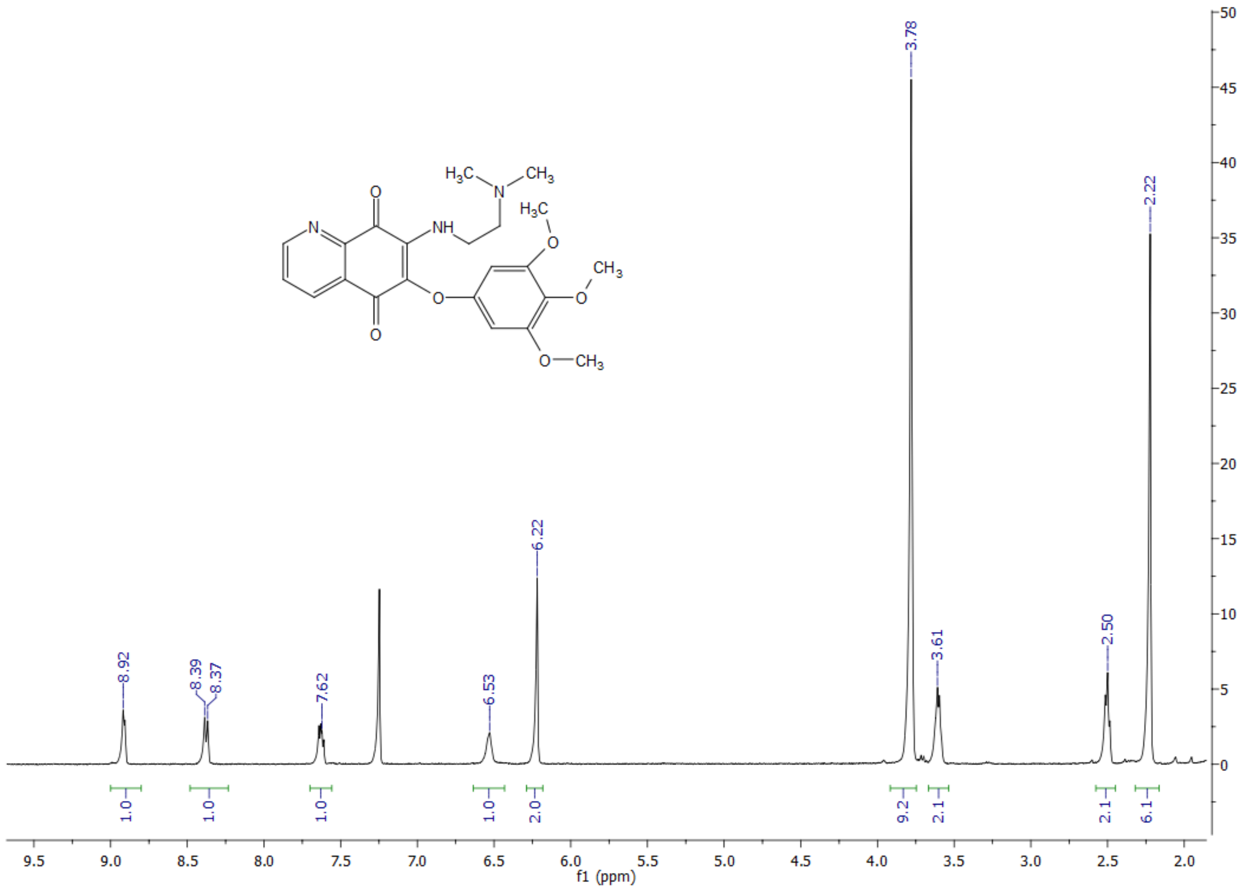

Figure S16. ^{1}HNMR spectrum (400MHz, $CDCl_3$) of compound **2c.**

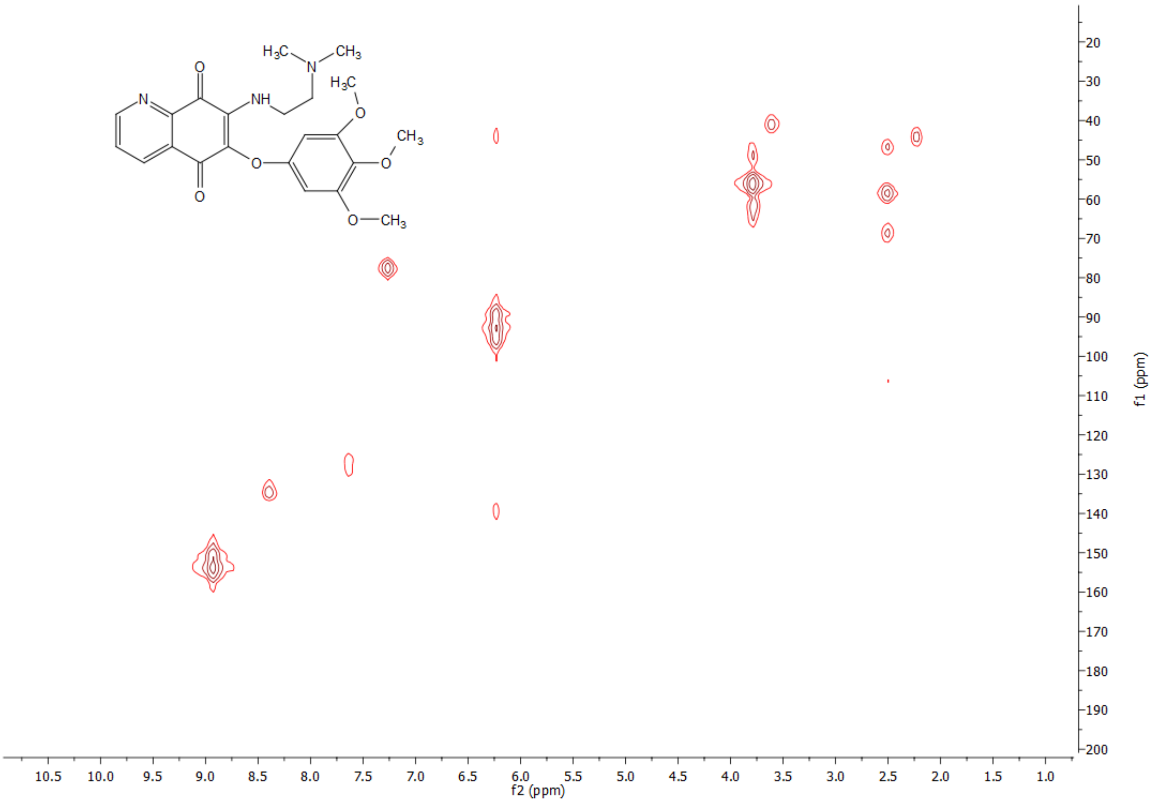

Figure S17. ${}^{1}H,{}^{13}C$ correlations by HSQC experiment (400MHz, $CDCl_3$) of **2c.**

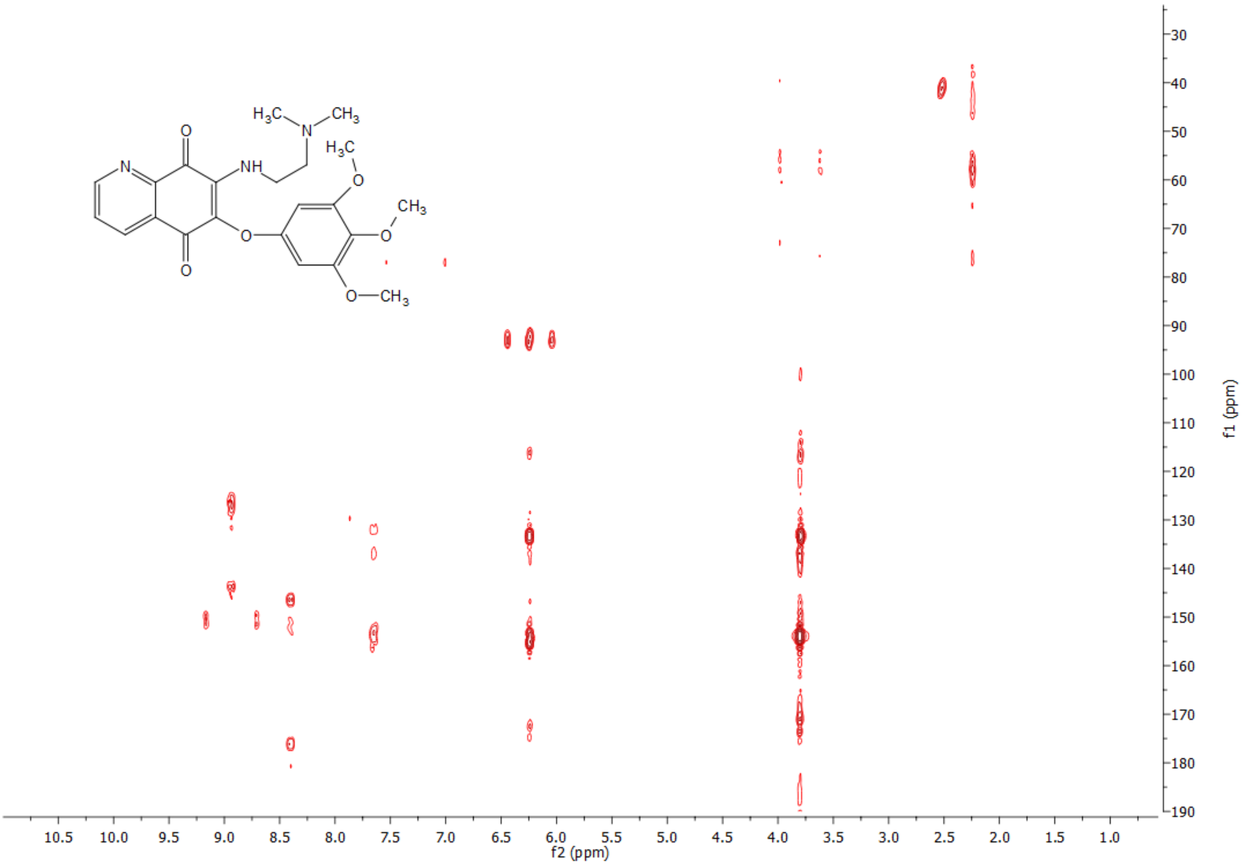

Figure S18. ${}^{1}H,{}^{13}C$ long range correlations by HMBC experiment (400MHz, $CDCl_3$) of **2c.**

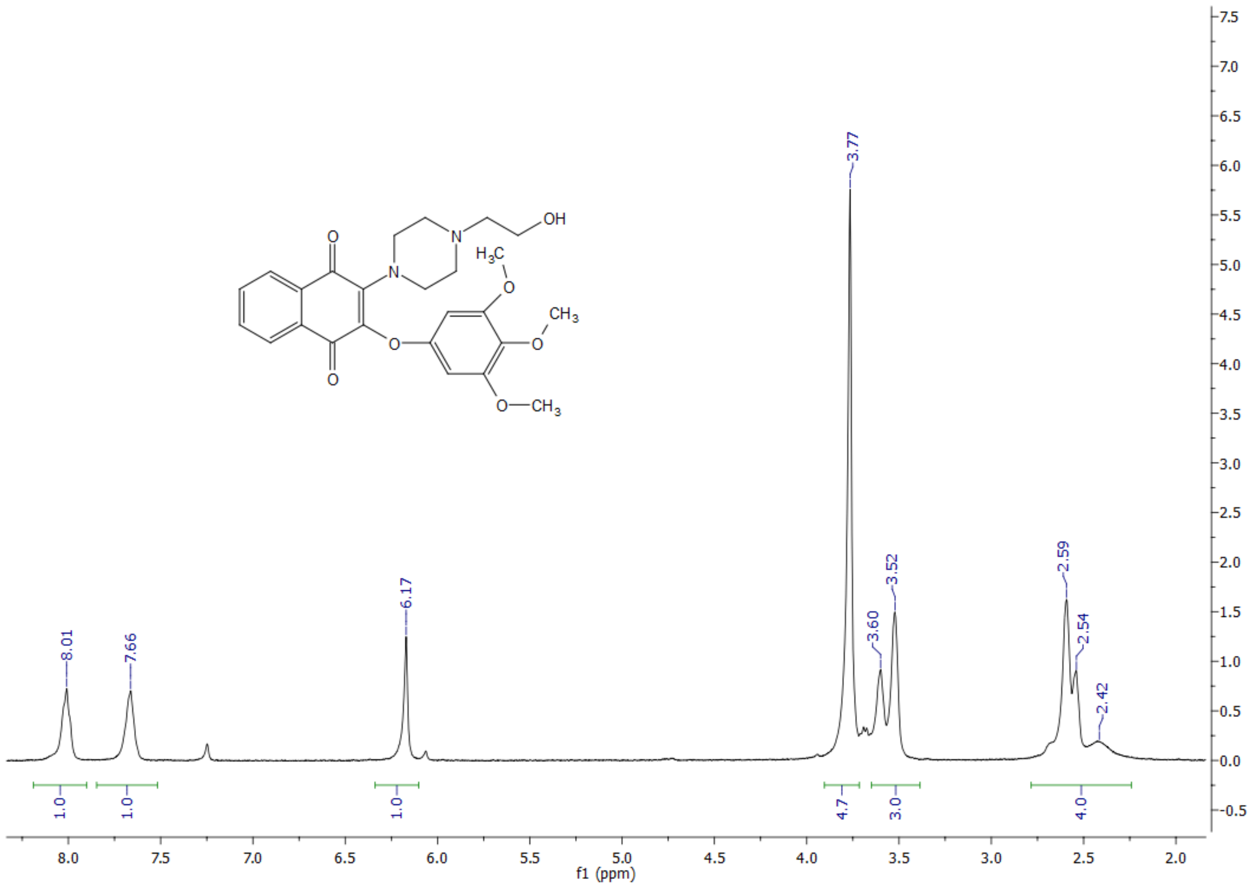

Figure S19. ^{1}HNMR spectrum (400MHz, $CDCl_3$) of compound **3.**

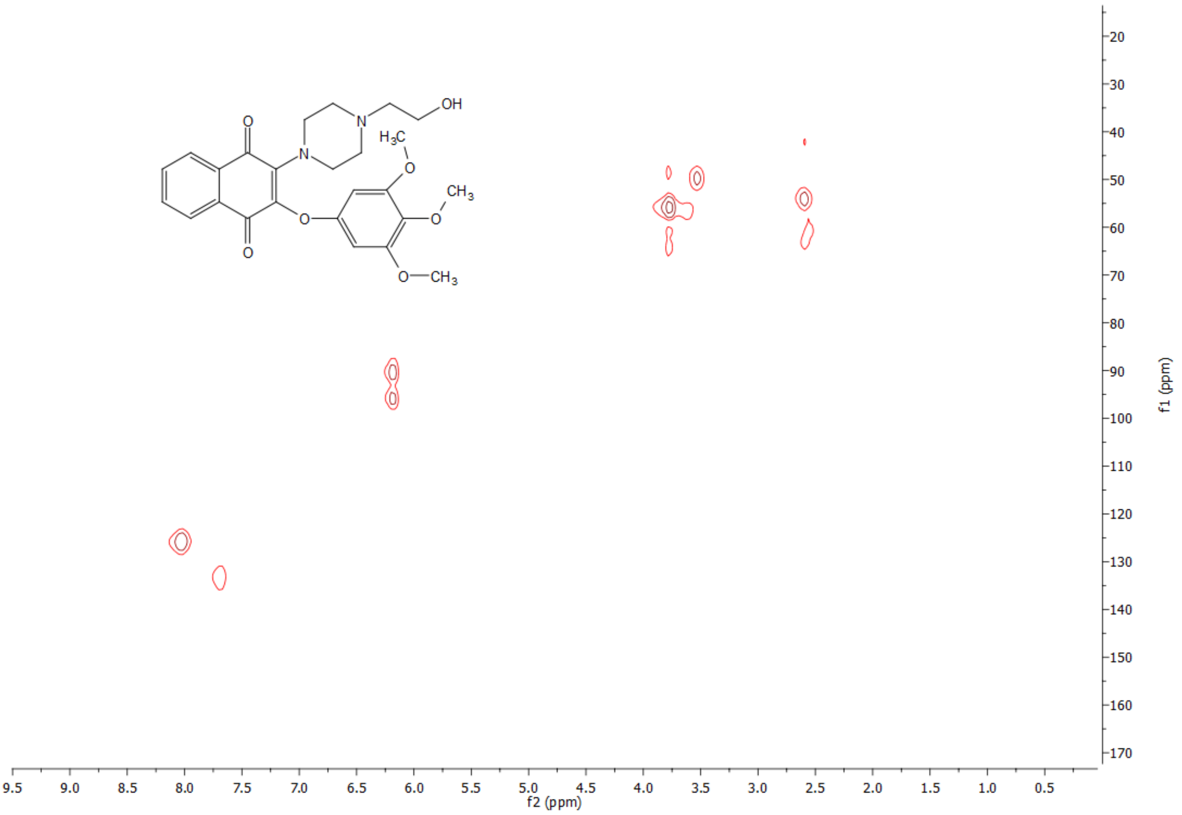

Figure S20. ^{1}H,^{13}C correlations by HSQC experiment (400MHz, $CDCl_3$) of **3.**

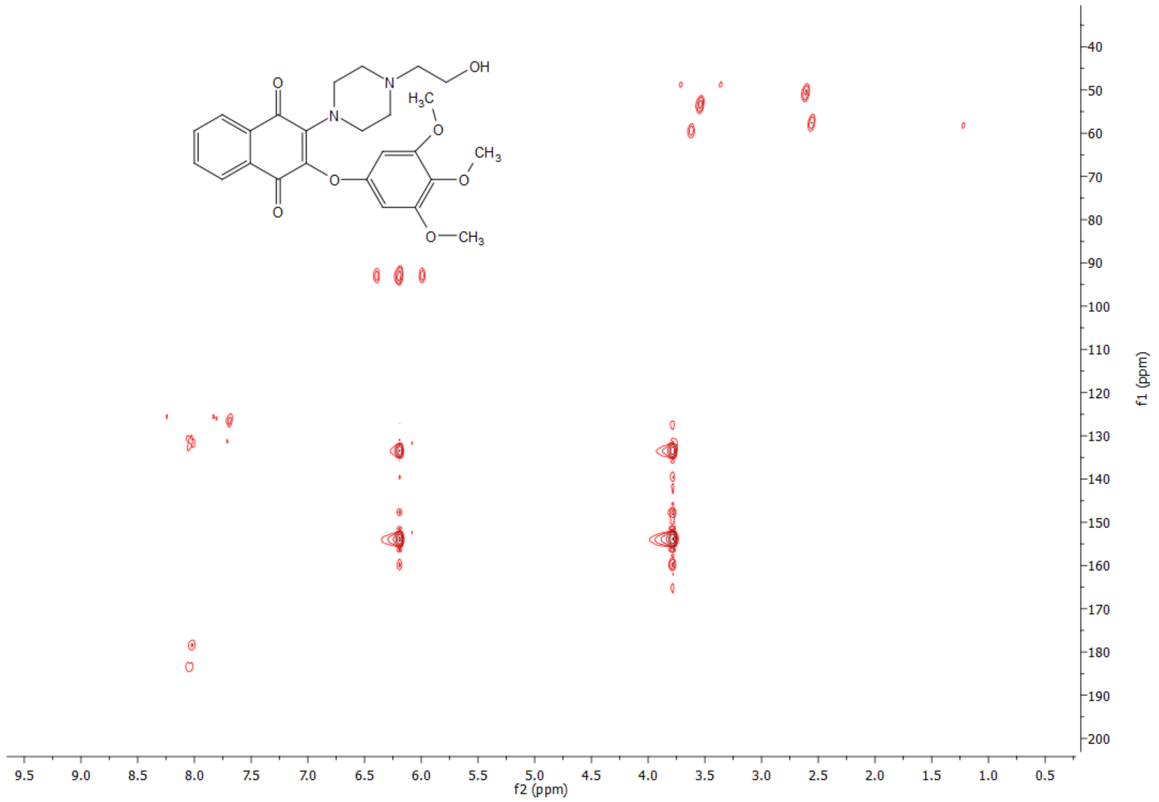

Figure S21. ^{1}H,^{13}C long range correlations by HMBC experiment (400MHz, $CDCl_3$) of **3.**

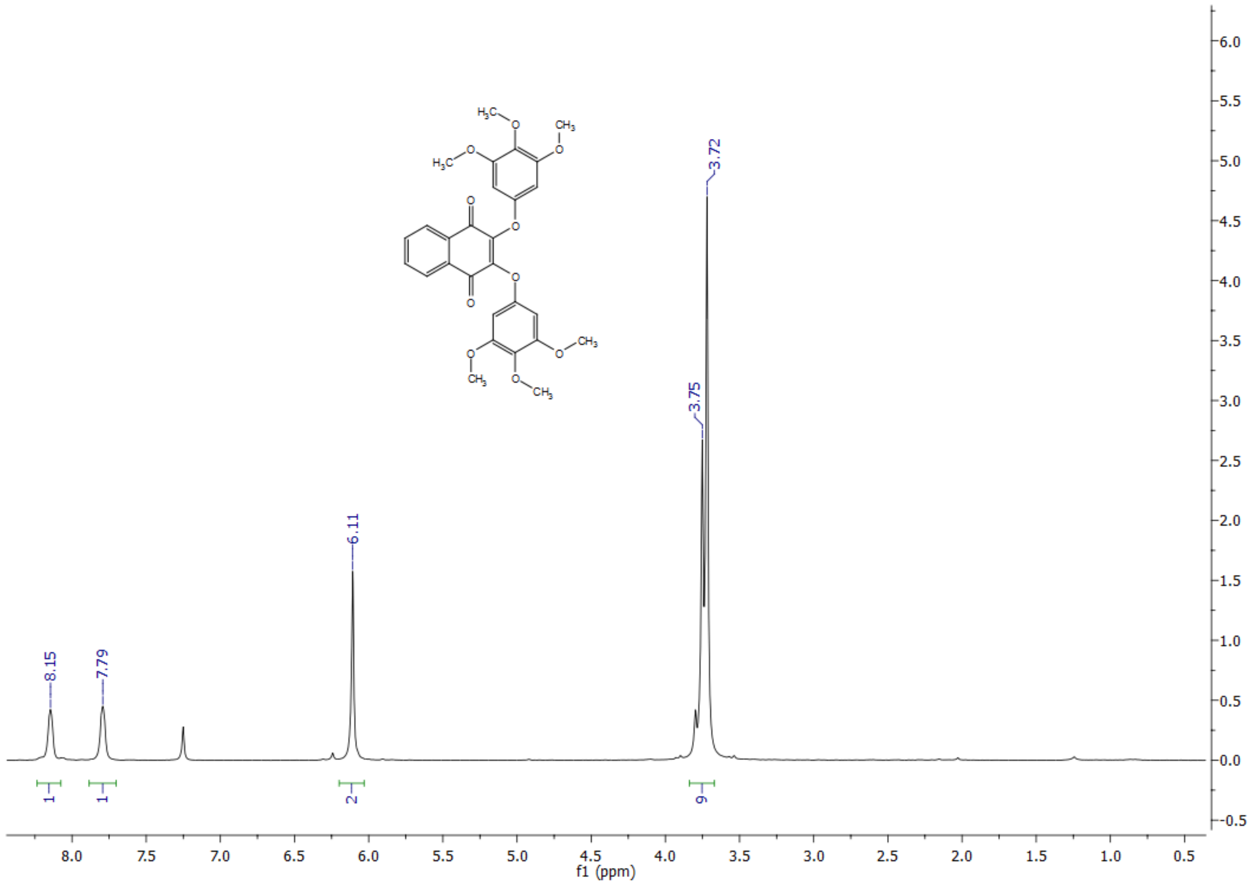

Figure S22. ^{1}HNMR spectrum (400MHz, $CDCl_3$) of precursor **4.**

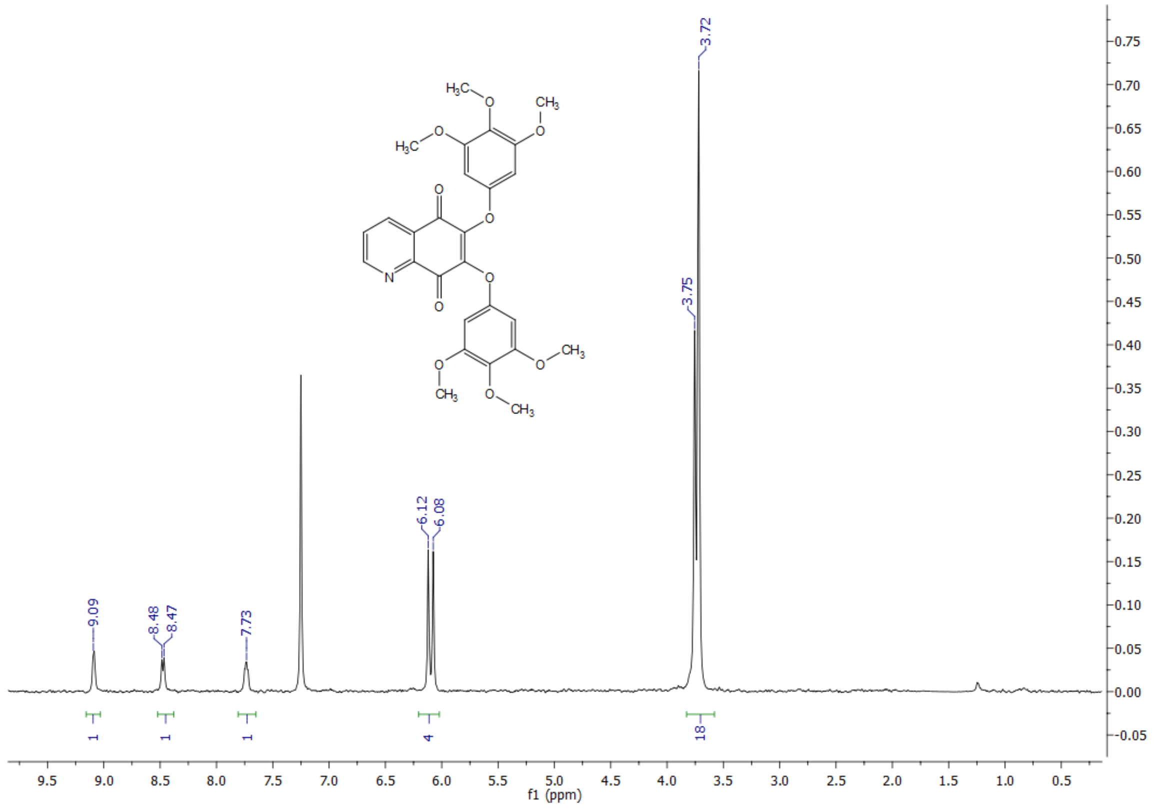

Figure S23. ^{1}HNMR spectrum (400MHz, $CDCl_3$) of precursor **5.**
